# Persistent left superior vena cava: clinical importance and differential diagnoses

**DOI:** 10.1186/s13244-020-00906-2

**Published:** 2020-10-15

**Authors:** Aynur Azizova, Omer Onder, Sevtap Arslan, Selin Ardali, Tuncay Hazirolan

**Affiliations:** Department of Radiology, Hacettepe University School of Medicine, 06100 Ankara, Turkey

**Keywords:** Persistent left superior vena cava, Cardiac anomalies, Clinical significance, Differential diagnoses, Computerized tomography

## Abstract

Persistent left superior vena cava (PLSVC) is the most common thoracic venous anomaly and may be a component of the complex cardiac pathologies. While it is often asymptomatic, it can lead to significant problems such as arrhythmias and cyanosis. Besides, it can cause serious complications during vascular interventional procedures or the surgical treatment of cardiac anomalies (CA). The clinical significance of PLSVC depends on the drainage site and the accompanying CA. In this article, we will describe the epidemiology, embryology, and anatomic variations of PLSVC. Possible accompanying CA and heterotaxy spectrum will be reviewed with the help of multidetector computed tomography (MDCT) images. Radiological pitfalls, differential diagnoses, and the clinical importance of PLSVC will be highlighted.

## Key points

- Persistent left superior vena cava (PLSVC) may lead to significant clinical symptoms and may affect surgical management.
- PLSVC may accompany various congenital cardiac diseases as well as heterotaxy spectrum.
- To be aware of the differential diagnoses of PLSVC is essential for correctly interpreting left-sided mediastinal vascular structures.

## Background

Persistent left superior vena cava (PLSVC) is a rare vascular anomaly that begins at the junction of the left subclavian and internal jugular veins, passes through the left side of the mediastinum adjacent to the arcus aorta. It mostly drains into the right atrium via the coronary sinus (CS). Although PLSVC is infrequent among all vascular anomalies, it is the most common thoracic venous anomaly. Mostly, PLSVC is asymptomatic and detected incidentally in diagnostic and therapeutic examinations due to different reasons. However, it can be discovered as a component of the complex cardiac pathologies and may lead to significant problems such as arrhythmia [1–4].

There are different modalities for evaluation of PLSVC, such as perinatal echocardiography, multidetector computed tomography (MDCT), magnetic resonance imaging (MRI), and invasive angiography. The advantages, disadvantages of these modalities, and optimal techniques for imaging of PLSVC are shown in Table 1 [5–7].

**Table 1:** The advantages, disadvantages, and techniques of different modalities for evaluation of PLSVC

Imaging modality	Pros	Cons	Techniques
Echocardiography	✓ Cheap ✓ Widely available ✓ No ionizing radiation ✓ Not affected by cardiac rhythm ✓ Portable (bedside assessment) ✓ Real-time imaging ✓ Enables evaluation of flow direction	Difficult to interpret Operator-dependent Acoustic window dependent The spatial resolution could be limited.	* Coexistence of dilated coronary sinus without any evidence of right-sided congestion and positive “Bubble study” are diagnostic sonographic findings for PLSVC. * “Bubble study” is conducted with the injection of agitated saline from the left peripheral arm veins. If PLSVC is present, the agitated saline bubbles firstly are seen in the coronary sinus, before the right atrium. * In case of isolated PLSVC, positive “Bubble study” is observed after injection from right peripheral arm veins, as well. * Contrast-enhanced echocardiography and transesophageal echocardiography are other useful modalities for the detection of PLSVC.
Multidetector computed tomography	✓ Accessible ✓ Fast scanning speed ✓ The best spatial resolution ✓ Enables multiplanar imaging and reformatting	Radiation exposure (Recently developed dose reduction methods have partially reduced concerns about radiation exposure.) An iodinated contrast agent (allergy, nephrotoxicity) Cardiac rhythm changes may cause artifacts. Need for sedation in the pediatric age group	* “ECG-gated CCTA with thin slices and multiplanar reformation” provides a detailed assessment. * “Intravenous non-ionic iodinated contrast injection with a dose of 0,5-2 ml/kg at a rate of 1-2 ml/s” is recommended. *The identification of PLSVC is usually independent of the contrast injection route (right or left, upper or lower extremities). The optimal contrast opacification of PLSVC is mostly seen in the delayed venous phase images.
Magnetic resonance imaging	✓ Radiation free ✓ High spatial resolution ✓ Enables multiplanar image acquisition ✓ Enables assessment of flow direction ✓ Depiction of PLSVC even without the administration of contrast media ✓ Non-iodinated contrast	High cost Less accessible Slow scanning speed Contraindications such as the magnetic implant, claustrophobia. Cardiac rhythm changes may cause artifacts. Need for sedation in the pediatric age group	* Axial and coronal cine SSFP sequences are the best sequences for imaging of PLSVC. * The black blood TSE T2 images is also useful. * Contrast-enhanced MRA (± Dynamic imaging) and phase contrast angiography can be used as auxiliary modalities.
Invasive angiography	✓ Gold standard ✓ Excellent morphologic information ✓ Interventions can be made if necessary	Invasive Radiation exposure An iodinated contrast agent (Allergy, nephrotoxicity) Need for sedation in the pediatric age group	* The catheter angiography with water-soluble contrast agent is performed. Venograms are obtained after bolus contrast injection from the catheter. * Invasive angiography is not a routine imaging modality for evaluation PLSVC. * PLSVC can be detected incidentally during procedures like central venous catheter insertion or pacemaker implantation.

*PLSVC* persistent left superior vena cava, *ECG*-*gated CCTA* electrocardiogram-gated coronary computed tomography angiography, *SSFP* steady-state free precession, *TSE* turbo spin echo, *MRA* magnetic resonance angiography

In this article, we will describe the epidemiology, embryology, and anatomic variations of PLSVC. Possible accompanying cardiac anomalies (CA) and heterotaxy spectrum will be reviewed with the help of MDCT images. The radiological pitfalls with their CT imaging features that may help make the differential diagnosis, and the clinical importance of PLSVC will be highlighted.

## Epidemiology

The exact frequency of PLSVC is not known because PLSVC is often asymptomatic and is detected incidentally. There is no significant difference in its prevalence between males and females. The prevalence of PLSVC ranges from 0.2 to 3% in the general healthy population. In patients with congenital heart disease (CHD), its prevalence ranges between 1.3 and 11%. Additionally, the prevalence of PLSVC is thought to be higher in the prenatal period since the accompanying anatomic anomalies, including heart defects, may cause spontaneous abortions and premature deaths [1–3, 8, 9].

## Embryology

**Fig. 1:**
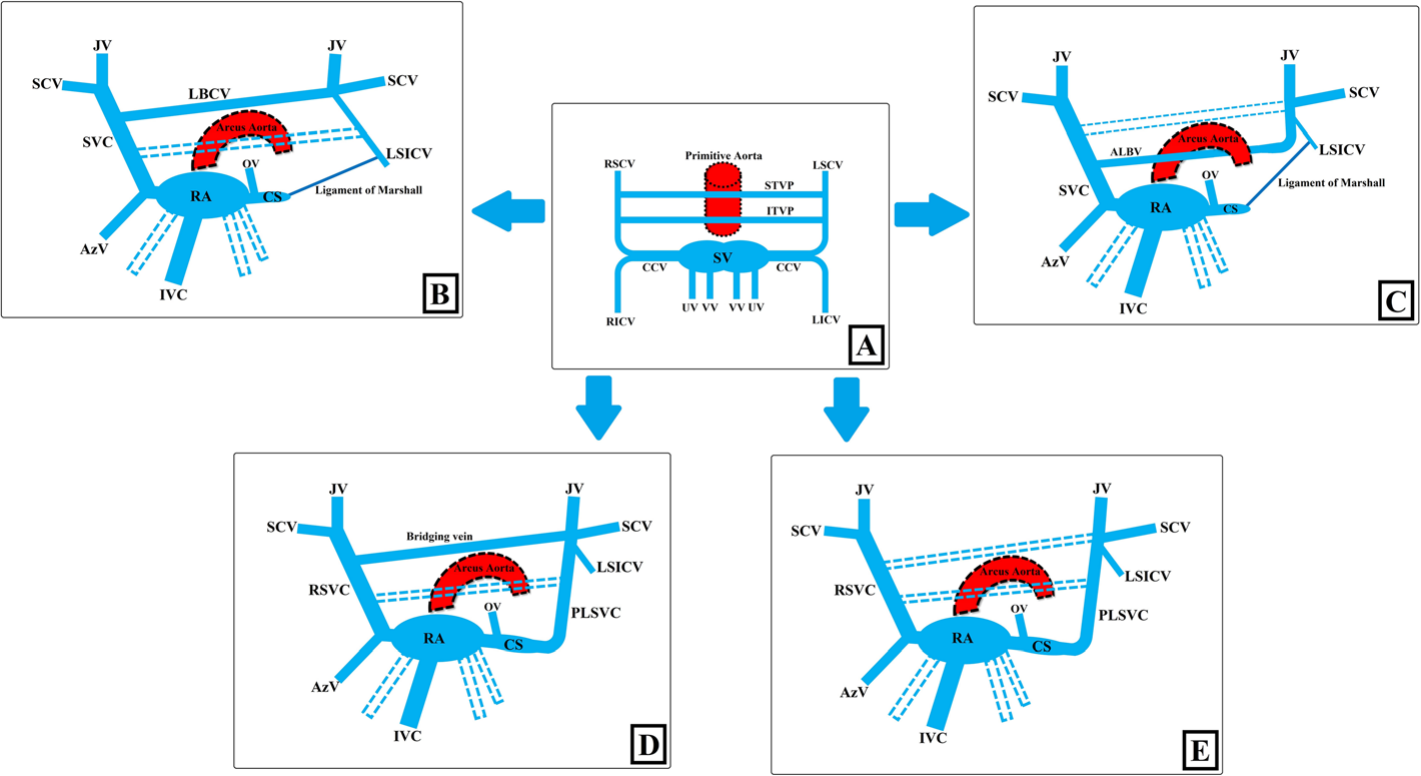
Developmental stages of primitive venous system and embryology of PLSVC. **a** In the 5th week of the intrauterine life, there are three paired veins: vitelline veins (VV), umbilical veins (UV), cardinal veins. Right and left superior and inferior cardinal veins ( respectively, RSCV, RICV, LSCV, LICV) combine to form common cardinal veins (CCV) draining into the double horned sinus venosus (SV). The sinus venosus accepts blood, also, from UVs and VVs. Transverse venous canals, called superior and inferior transverse venous plexus (respectively, STVP and ITVP), develop around the primitive aorta and connect RSCV and LSCV. **b** In the 8th week, the cranial parts of RSCV and LSCV form internal jugular (JV), subclavian (SCV), and brachiocephalic veins. The caudal part of RSCV, together with the right CCV, forms the superior vena cava (SVC). The caudal part of LSCV firstly forms the left superior intercostal vein (LSICV). Then, together with the left CCV, it transforms into the Marshall ligament. RICV forms azygos vein (AzV), and LICV regresses. While the left VV with right and left UV regresses (dashed lines), the right VV forms inferior vena cava (IVC). During this period, the right horn of the sinus venosus joins into the right atrial structure (RA) while the left horn turns into the oblique vein of the left atrium (OV) and the coronary sinus (CS), which drains major cardiac veins. The STVP contributes to the formation of the left brachiocephalic vein (LBCV) while the ITVP regresses (dashed lines) with the compression by the growing aorta and pulmonary artery. **c** If the STVP regresses (dashed lines) by the highly located aortic arch, such as the right aortic arch, the cervical aortic arch, the ITVP continues to develop and turns into the aberrant left brachiocephalic vein (ALBV). **d, e** If the left CCV and the caudal part of LSCV do not regress, they will persist as PLSVC. There could be LBCV connecting right SVC (RSVC) and PLSVC, which is also called the bridging vein (**d**). The STVP, which forms the LBCV, may undergo idiopathic regression (dashed lines) in PLSVC existence, resulting in the absence of the bridging vein (**e**)

The primitive venous system consists of three paired veins: vitelline veins (VV), umbilical veins (UV), cardinal veins (CV). Superior and inferior CVs are essential structures that allow the blood to return from the cranial and caudal parts of the embryo to the primitive heart. They combine to form common CVs (or the duct of Cuvier) draining into the double horned sinus venosus [2, 3, 9]. The caudal part of the right superior CV, together with the common CV, forms the right superior vena cava (RSVC). Generally, the left common CV and the caudal part of the left superior CV will regress. If these veins do not regress, then they will persist as PLSVC [2, 3, 8–11]. The detailed schematic anatomy of the developmental stages of the primitive venous system is shown in Fig. 1.

Different hypotheses regarding the development of PLSVC have been proposed. One of these is “low left atrial pressure theory.” According to this theory, in the presence of anomalies, which may cause reduced left atrial pressure and insufficient development of the left atrium, such as atrioventricular septal defect (AVSD), the left atrium will be smaller than expected. Thus, it will not be able to compress the CS and left CVs adequately. As such, the left common CV and caudal part of the left superior CV will not regress, and PLSVC will develop. Some hypotheses suggest the vice versa. According to the “obstructive theory” hypothesis, the presence of PLSVC, which may cause an increase in CS size, could lead to the formation of a left-sided obstructive lesion because of the space restriction [1].

## Drainage site and its impact on the anatomy

**Fig. 2:**
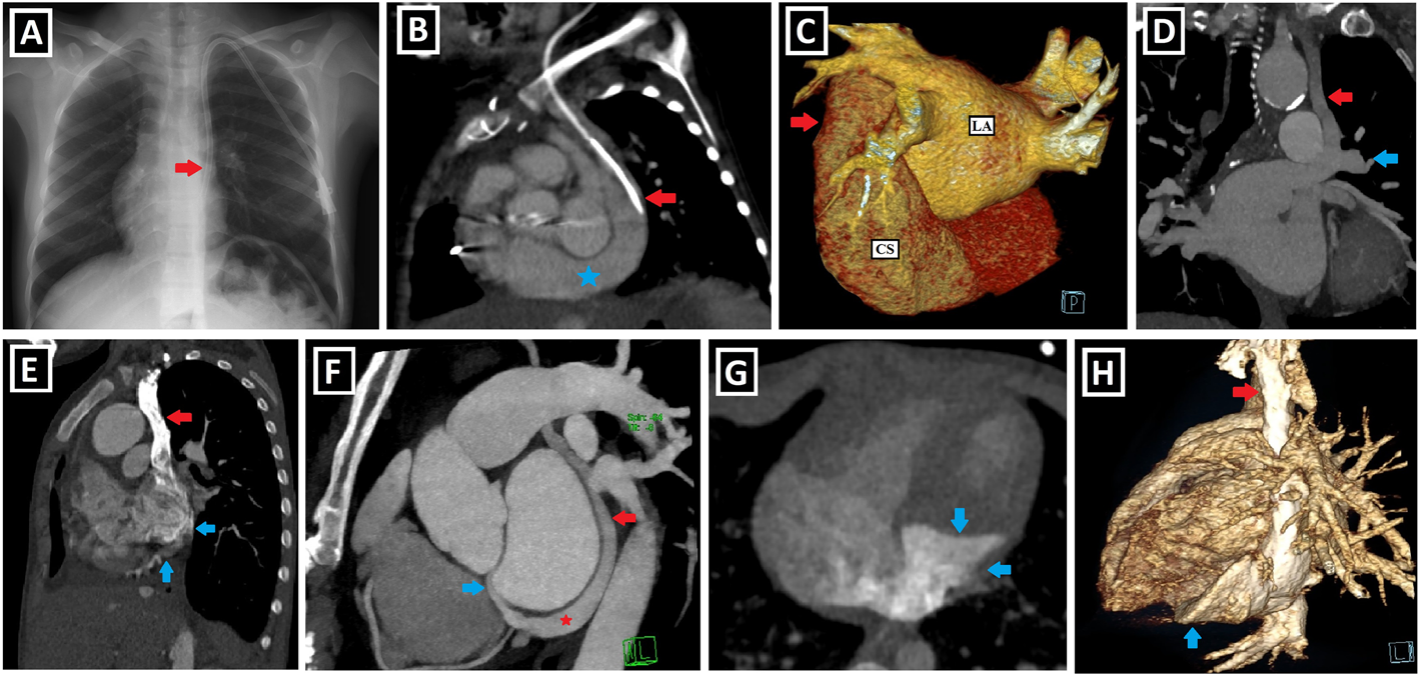
Clinical importance and different drainage sites of PLSVC and possible anatomical changes. **a, b** Posterior-anterior (PA) chest X-ray (**a**), and sagittal-oblique reformatted CT image (**b**) of different patients depict the course of central venous catheters inserted into the PLSVC (red arrows) draining into the right atrium via the coronary sinus (blue star). **c** CT imaging performed to evaluate the cardiac anatomy and possible variations of pulmonary veins draining to the left atrium (LA) before the radiofrequency catheter ablation in a patient with atrial fibrillation. The three-dimensional volume rendering technique (3D VRT) image shows that PLSVC (red arrow), which is detected incidentally, drains to the coronary sinus (CS). **d** Coronal-oblique maximum intensity projection CT image indicates PLSVC (red arrow), which indirectly drains into the left atrium via the left upper pulmonary vein (blue arrow). **e** Sagittal-oblique reformatted CT image shows PLSVC (red arrow) draining into the left atrium via the unroofed coronary sinus (blue arrows). Since ASD (not shown) is also present, the findings are compatible with Raghib syndrome. **f** Sagittal-oblique maximum intensity projection CT image demonstrates that the coronary sinus is connected to the left atrium with an aberrant vein as a collateral drainage pathway in a patient with coronary sinus ostial atresia (not shown). Thus, PLSVC (red arrow) drains into the left atrium through the coronary sinus (red star) and interatrial aberrant vein (blue arrow). **g**, **h** Axial CT (**f**) and 3D VRT images with different views (**g**, **h**) indicate the coronary sinus aneurysm (blue arrows) in a patient with PLSVC (red arrow) draining into the coronary sinus

PLSVC is responsible for approximately 20% of the total venous blood return from the left arm, left half of the head and neck. The right atrial drainage is seen in 80–90% of cases, while the left atrial drainage accounts for the remaining 10–20%. Generally, it joins into the right atrium through the CS and mostly has no hemodynamic effect. However, CS ostial atresia may accompany PLSVC. In that case, PLSVC becomes the major retrograde drainage pathway for coronary veins unless collateral drainage pathways develop between the coronary sinus and the heart chambers. The left atrial drainage, which is rare, occurs directly via the left atrial appendage or indirectly through the left pulmonary veins or the CS. In some sources, the latter is defined as an unroofed CS or CS atrial septal defect. The association of the atrial septal defect (ASD) and PLSVC draining into the left atrium via unroofed CS is called as Raghib syndrome (Fig. 2a–f) [2–4, 9, 12].

Left atrial drainage is a cause of right-to-left shunt and is mostly accompanied by CA. However, it was reported that this condition might also be observed without any cardiac defects [2, 3, 12].

It has been reported that the drainage of more than the expected venous blood volume into the right atrium leads to some changes in the heart anatomy. Of those, the most well-known is the enlargement of the CS, which is a helpful clue indicating PLSVC existence. This enlargement may rarely reach the aneurysmatic level (Fig. 2g, h). There are also other anatomical changes reported in the literature, and they are described in Table 2, together with possible underlying mechanisms [2, 3, 12].

**Table 2:** Anatomical changes and possible underlying mechanisms in the presence of PLSVC

Reported anatomical changes	Possible underlying mechanisms
The decrease in RSVC dimensions	Reduction of blood volume drained through RSVC
Decrease in mitral valve area	Compression to the left atrium via dilated CS
Atrophy of the valves of cardiac veins such as Vieussens, Thebesian	Increased blood volume draining into the CS
The presence of a common left pulmonary vein trunk	Limited space caused by the dilated CS
Increase in heart weight	–

*PLSVC* persistent left superior vena cava, *RSVC* right superior vena cava, *CS* coronary sinus

## Presence of RSVC and bridging vein

**Fig. 3:**
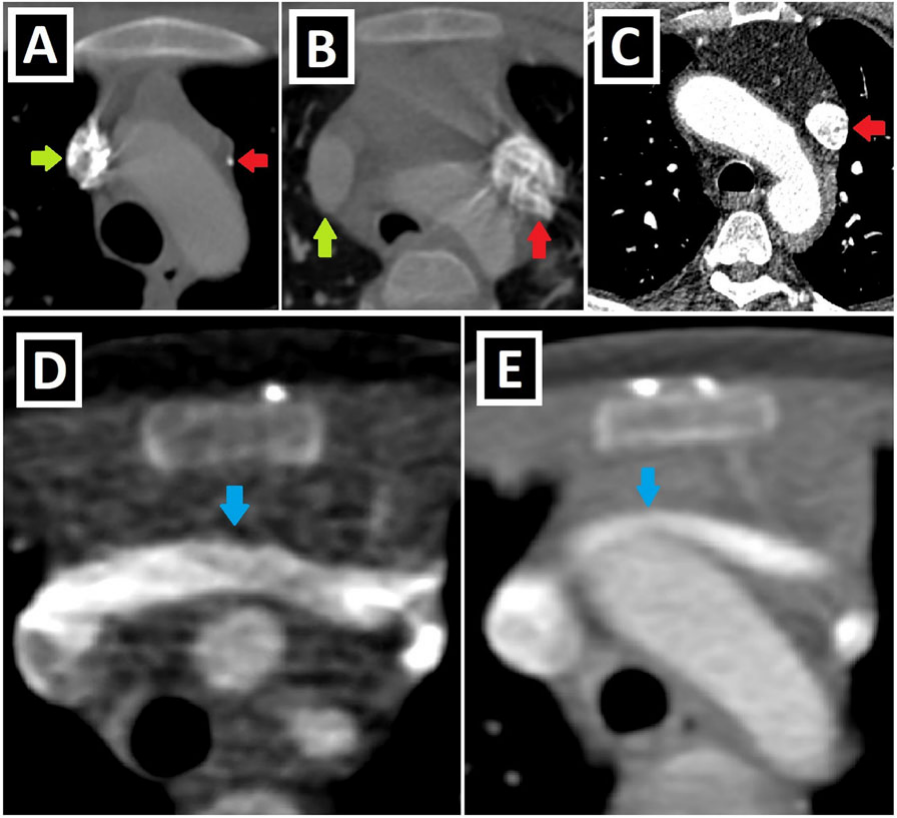
Size of PLSVC, presence of RSVC/bridging vein. Axial CT images of different patients. **a** PLSVC (red arrow) with a significantly smaller caliber than RSVC (green arrow). **b** PLSVC (red arrow) having similar sizes to RSVC (green arrow) in a patient with double SVC. **c** Isolated PLSVC (red arrow) in a patient without any accompanying cardiac anomaly. **d, e** Prominent (**d**) and relatively less prominent (**e**) bridging veins (blue arrows) compared to bilateral SVC diameters

In up to 90% of the cases, the right superior vena cava (RSVC) accompanies PLSVC, and this situation is known as double SVC (DSVC). If the caudal part of the right superior CV regresses in the intrauterine period, RSVC cannot develop, resulting in the presence of isolated PLSVC (IPLSVC). Mostly, IPLSVC is associated with CA and cardiac situs disorders. However, there are examples of IPLSVC without any accompanying apparent CA in the literature. In cases of DSVC, dimensions of RSVC may be larger or smaller than PLSVC (Fig. 3a–c) [2, 3, 9, 13].

In 65% of the cases, DSVC runs along each side of the mediastinum without interconnection. However, there could be the left brachiocephalic vein (LBCV) connecting them, which is also called the bridging vein (BV) (Fig. 3d, e) [3].

## PLSVC and accompanying cardiac anomalies

**Fig. 4:**
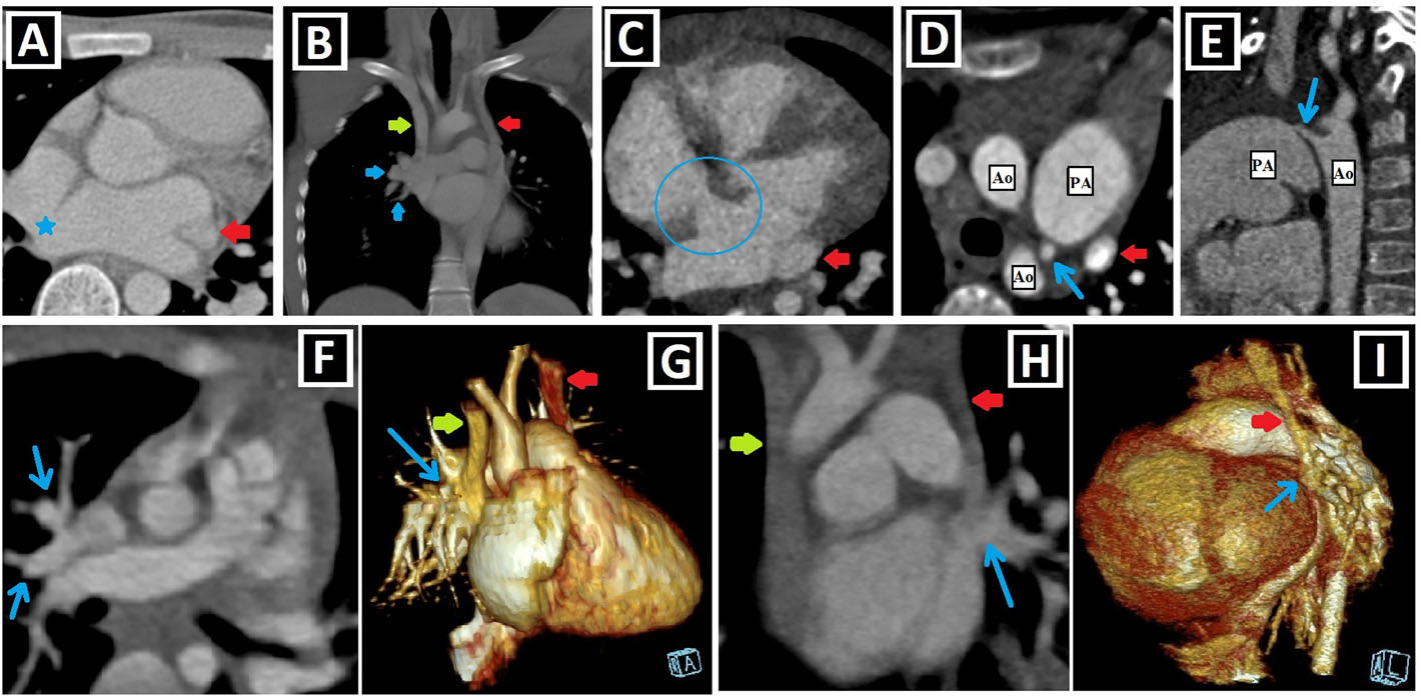
Shunt lesions accompanying PLSVC-1. **a, b** Axial (**a**) and coronal-oblique reformatted (**b**) CT images indicate **sinus** venosus ASD (blue star) in a patient with PLSVC (red arrow) and accompanying abnormal right upper pulmonary venous drainage (blue arrows) to RSVC (green arrow). **c** Axial CT image depicts the ostium secundum ASD (blue circle) in a patient with PLSVC (red arrow). **d, e** Axial (**d**) and sagittal-oblique reformatted (**e**) CT images depict the accompanying PDA (blue arrows) in a patient with PLSVC (red arrow). PA represents the pulmonary artery, and Ao represents aorta. **f, g** Axial (**f**) and 3D VRT (**g**) CT images indicate the PAPVD to the RSVC (blue arrows) in a patient with double SVC (green and red arrows). **h, i** Coronal-oblique reformatted (**h**), and 3D VRT reconstructed (**i**) CT images indicate the PAPVD to the PLSVC (blue arrows) in a patient with double SVC (green and red arrows)

**Fig. 5:**
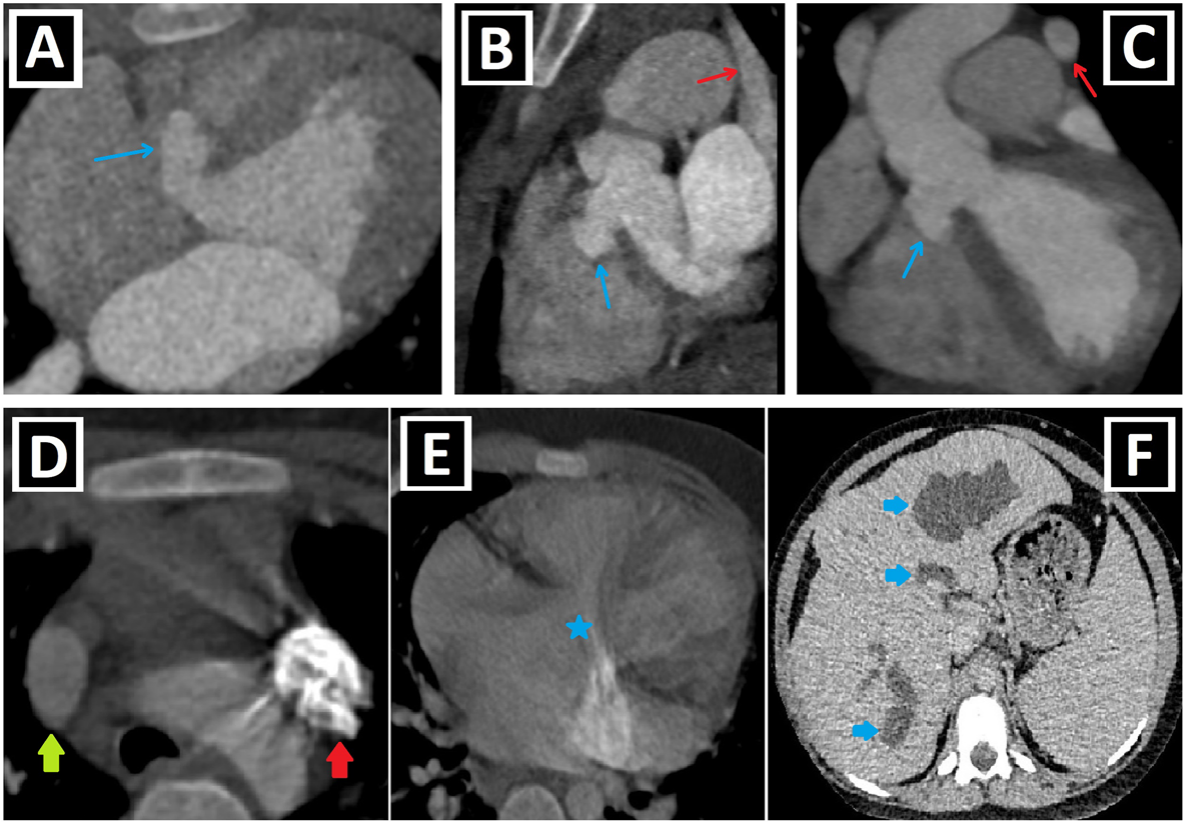
Shunt lesions accompanying PLSVC-2. **a–c** Axial (**a**), oblique sagittal (**b**), and oblique coronal (**c**) CT images depict interventricular membranous septal aneurysm (blue arrows) developing due to the spontaneous closure of VSD in a patient with PLSVC (red arrows). **d–f** Axial CT images indicate accompanying AVSD (blue star) in a patient with Caroli disease (blue arrows showing multifocal cystic dilatation of segmental intrahepatic bile ducts) and double SVC (green and red arrows).

**Fig. 6:**
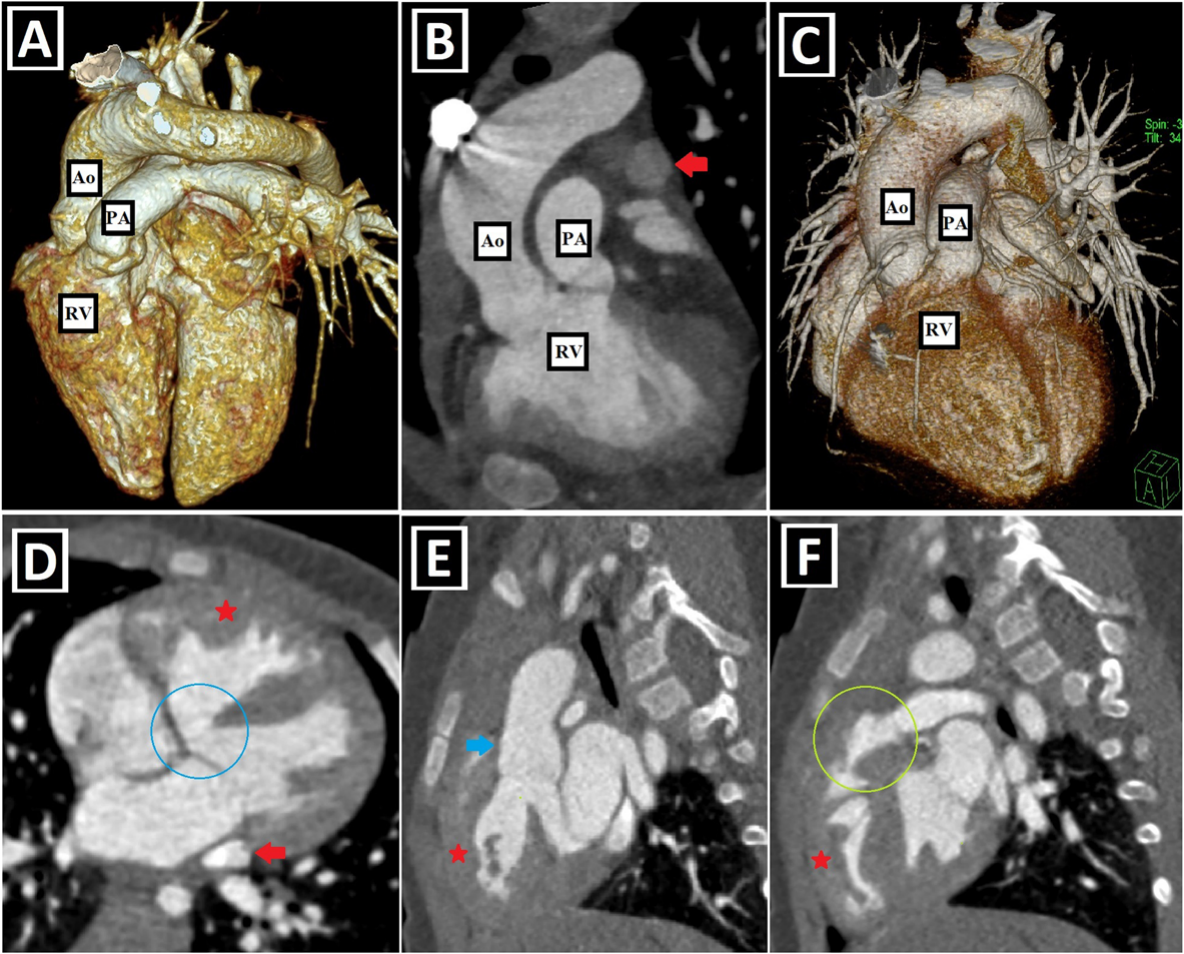
Conotruncal malformations accompanying PLSVC-1. **a–c** 3D VRT (**a**, **c**) and coronal-oblique reformatted (**b**) CT images show DORV characterized by great arteries (pulmonary artery: PA and aorta: Ao) arising primarily from the right ventricle (RV) in a patient with PLSVC (red arrow). **d–f** Axial (**d**) and sagittal-oblique reformatted (**e**, **f**) CT images depict TOF characterized by the combination of the right ventricular hypertrophy (red stars), subaortic VSD (blue circle), overriding aorta (blue arrow), and pulmonary stenosis (green circle) in a patient with PLSVC (red arrow)

**Fig. 7:**
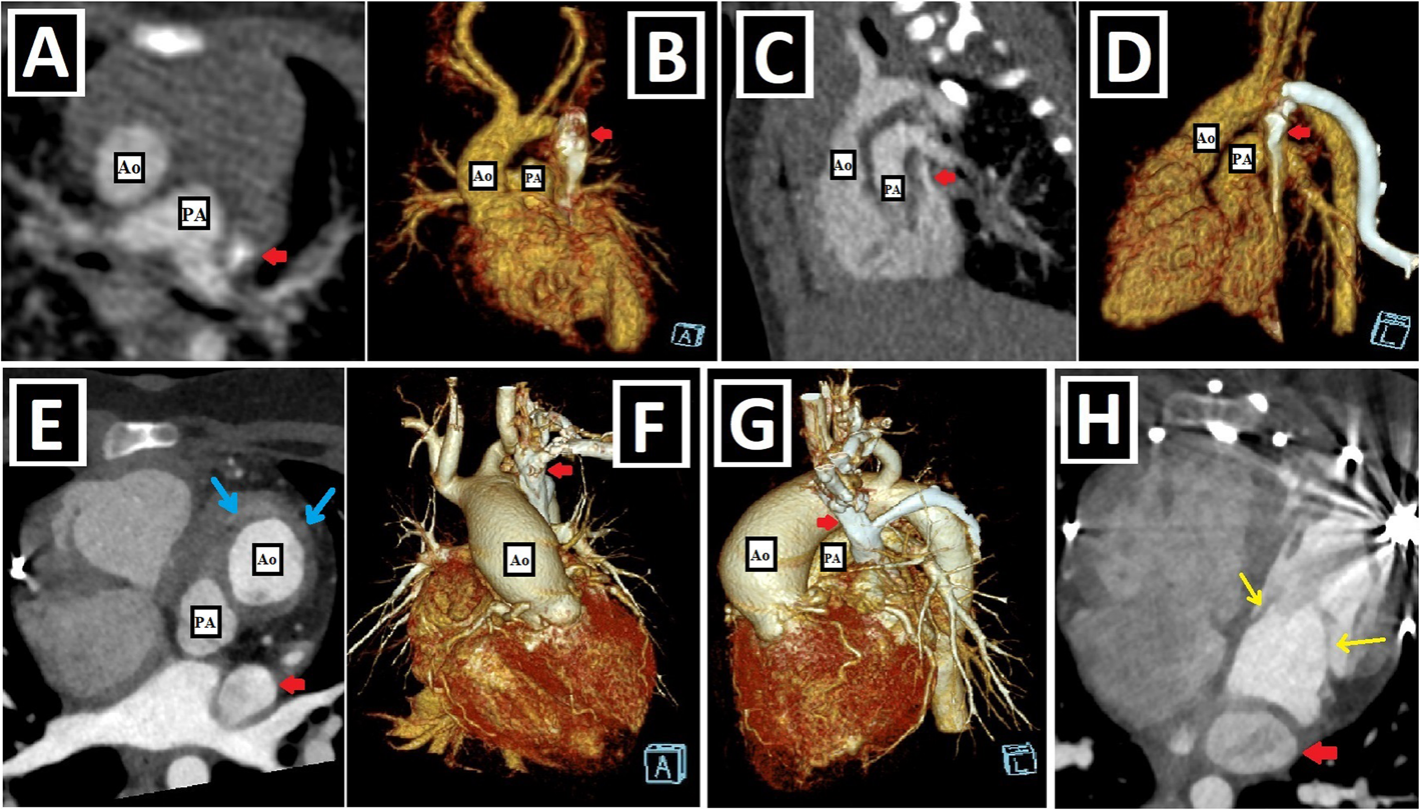
Conotruncal malformations accompanying PLSVC-2. **a–d** Axial (**a**), sagittal-oblique reformatted (**c**), and 3D VRT (**b**, **d**) CT images show the accompanying D-TGA in a patient with PLSVC (red arrows). The aorta (Ao) is located to the anterior and right of the pulmonary truncus (PA) (**a**, **b**). Please note the parallel course of the aorta and pulmonary truncus without “crossing over” (**c**, **d**)**. e–h** Axial (**e**, **h**) and 3D VRT (**f**, **g**) CT images depict the accompanying L-TGA anomaly in a patient with PLSVC (red arrows). The aorta (Ao) is located to the left and anterior of the pulmonary truncus (PA) (**e**–**g**). The infundibular muscle around the aorta (blue arrow) indicates the right ventricular origin (**e**). The left-sided ventricle has the tricuspid valve (yellow arrows), which is the closer atrioventricular valve to the ventricular apex and indicates the right ventricular configuration (**h**)

**Fig. 8:**
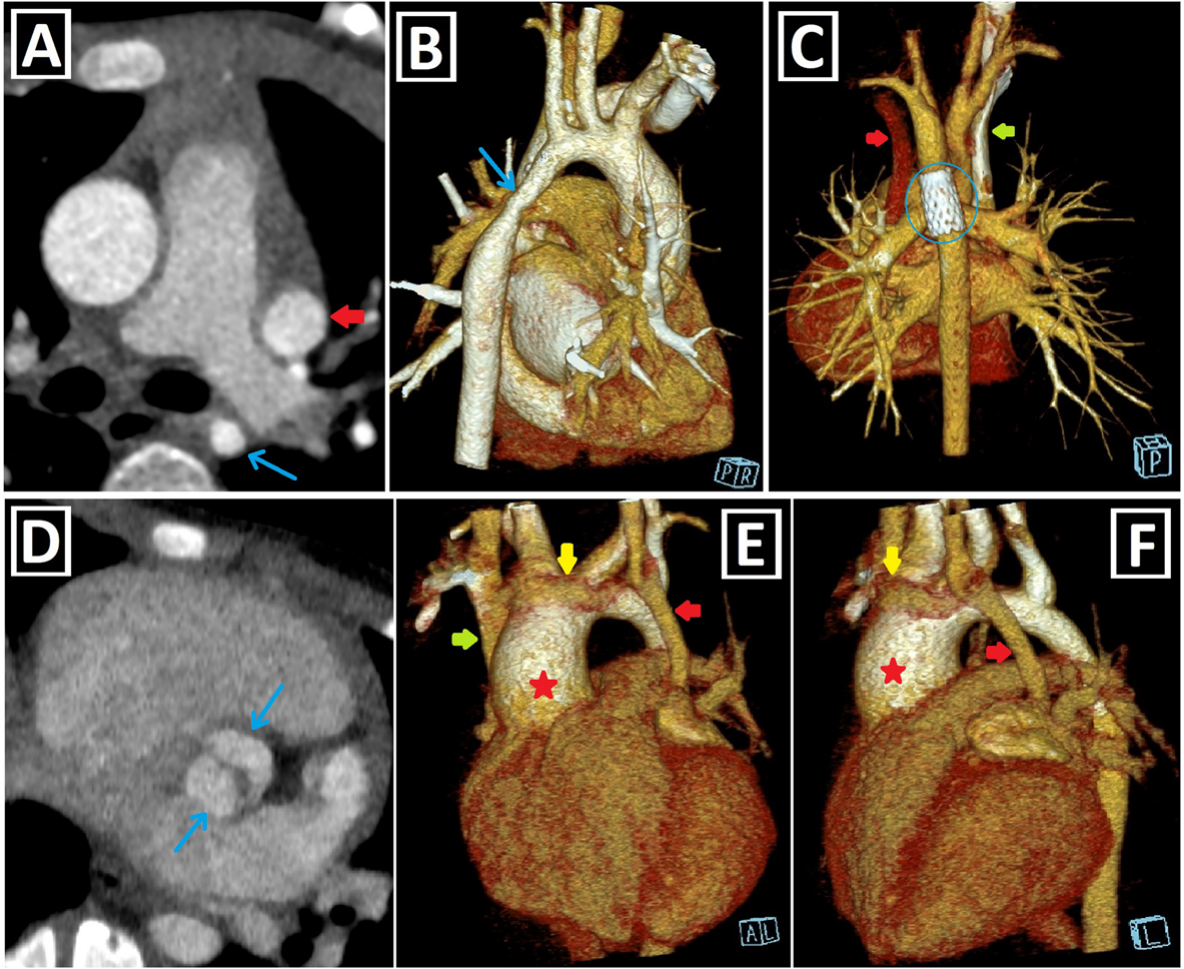
Left-sided obstructive lesions accompanying PLSVC. **a–c** Axial (**a**) and 3D VRT (**b**, **c**) CT images. The narrow segment compatible with aortic coarctation (blue arrows) is seen in a patient with PLSVC (red arrow) (**a**, **b**). Aortic coarctation treated by the endovascular intervention (blue circle) is depicted in another patient with double SVC (green and red arrows) (**c**). **d**–**f** Axial (**d**) and 3D VRT (**e**, **f**) CT images show the bicuspid aortic valve (blue arrows) (**d**) and the accompanying ascending aorta dilation (red stars) (**e**, **f**) in the patient with double SVC (green and red arrows). A bridging vein between RSVC and PLSVC (yellow arrows) is also seen (**e**, **f**)

**Fig. 9:**
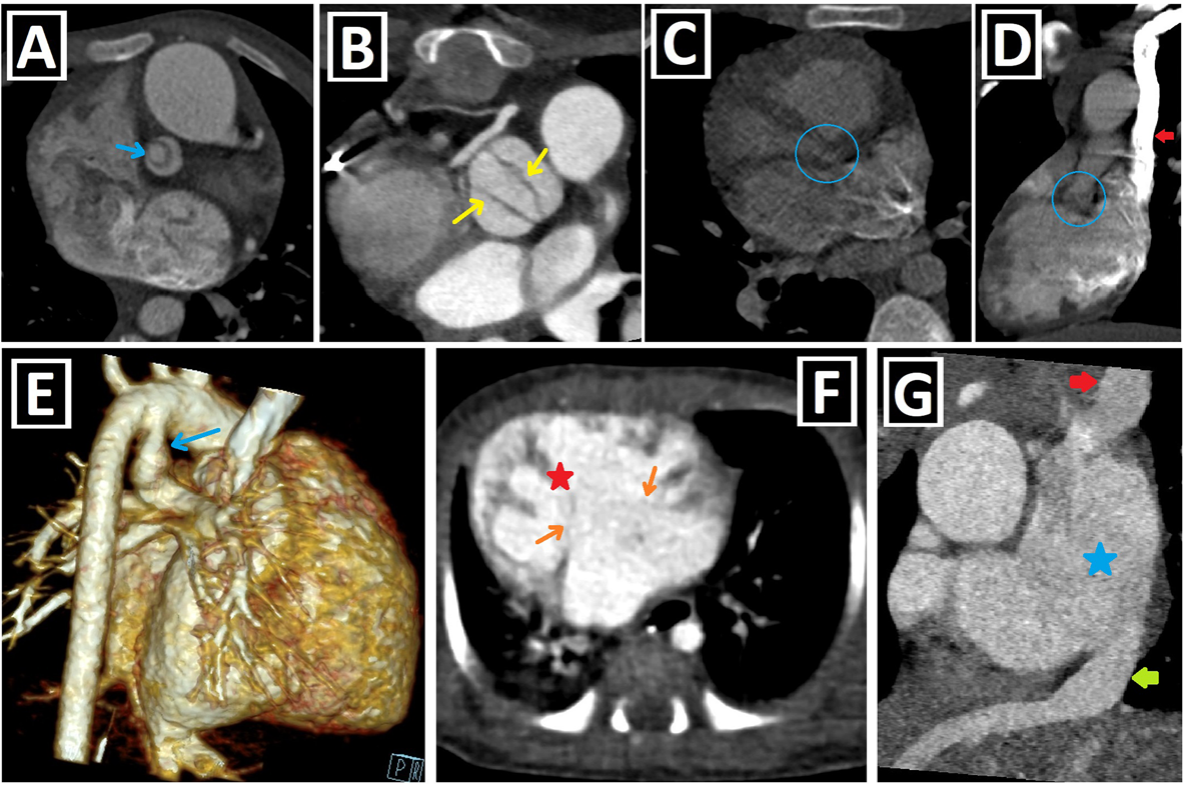
Right-sided lesions and single ventricular anomalies accompanying PLSVC. **a**, **b** Axial-oblique CT images indicate unicuspid (blue arrow, **a**) and bicuspid (yellow arrows, **b**) pulmonary valve in different patients with PLSVC. **c, d** Axial (**c**) and coronal-oblique reformatted (**d**) CT images depict severe pulmonary stenosis (blue circles) in a patient with PLSVC (red arrow). **e–g** 3D VRT (**e**), axial (**f**), and sagittal-oblique reformatted (**g**) CT images depict complex cardiac anomaly in a patient with PLSVC. This patient has pulmonary atresia with confluent right and left pulmonary arteries connected to the aorta with a large caliber PDA (blue arrow) (**e**). There is a single ventricle (red star) and a single atrioventricular valve (orange arrows) (**f**). PLSVC (red arrow) and IVC (green arrow) are draining into the common atrium (blue star) (**g**)

**Fig. 10:**
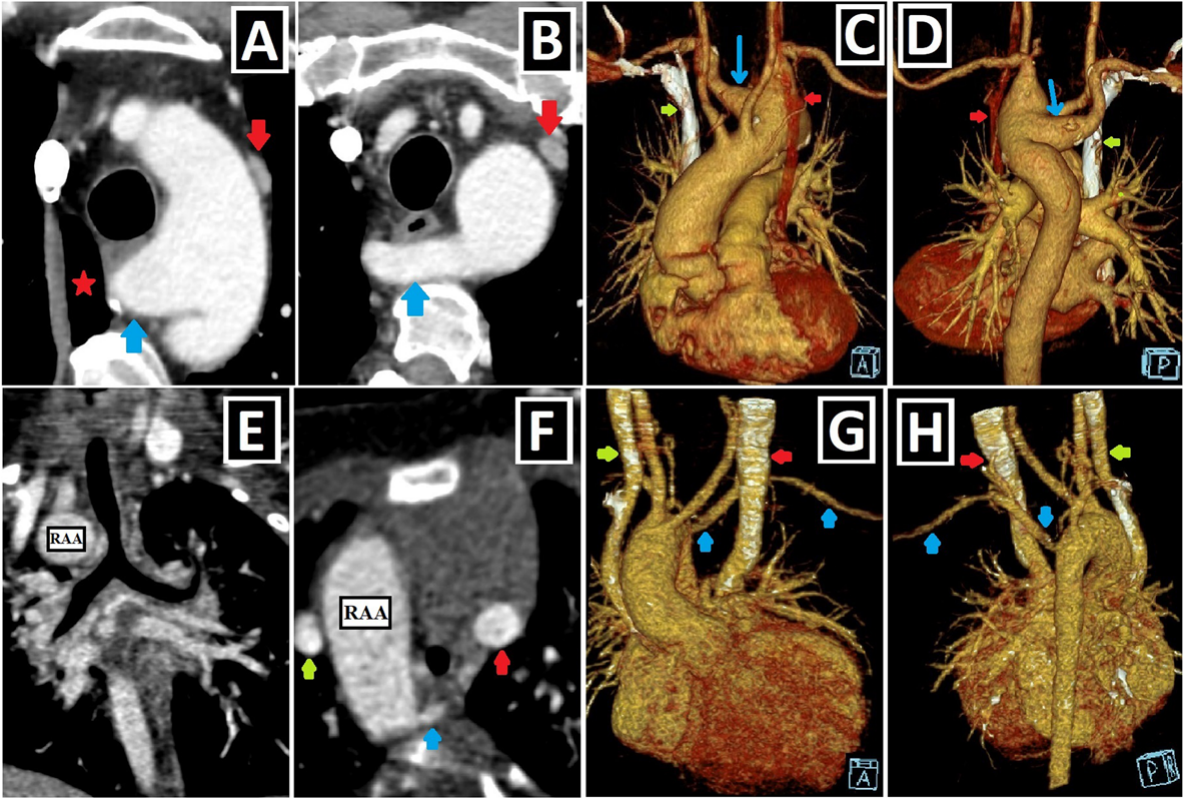
Aortic arch anomalies accompanying PLSVC-1. **a** Axial CT image depicts azygos lobe (red star), and ARSA with Kommerell diverticulum (blue arrow) in a patient with PLSVC (red arrow). **b** Axial CT image indicates ARSA (blue arrow) in a patient with PLSVC (red arrow). **c**, **d** 3D VRT images with anterior and posterior views depict ARSA with Kommerell diverticulum (blue arrows) in a patient with double SVC (green and red arrows). **e**–**h** Coronal-oblique reformatted (**e**), axial (**f**), and 3D VRT CT images with different views (**g**, **h**) show the right aortic arch (RAA) and ALSA (blue arrows) in a patient with double SVC (green and red arrows)

**Fig. 11:**
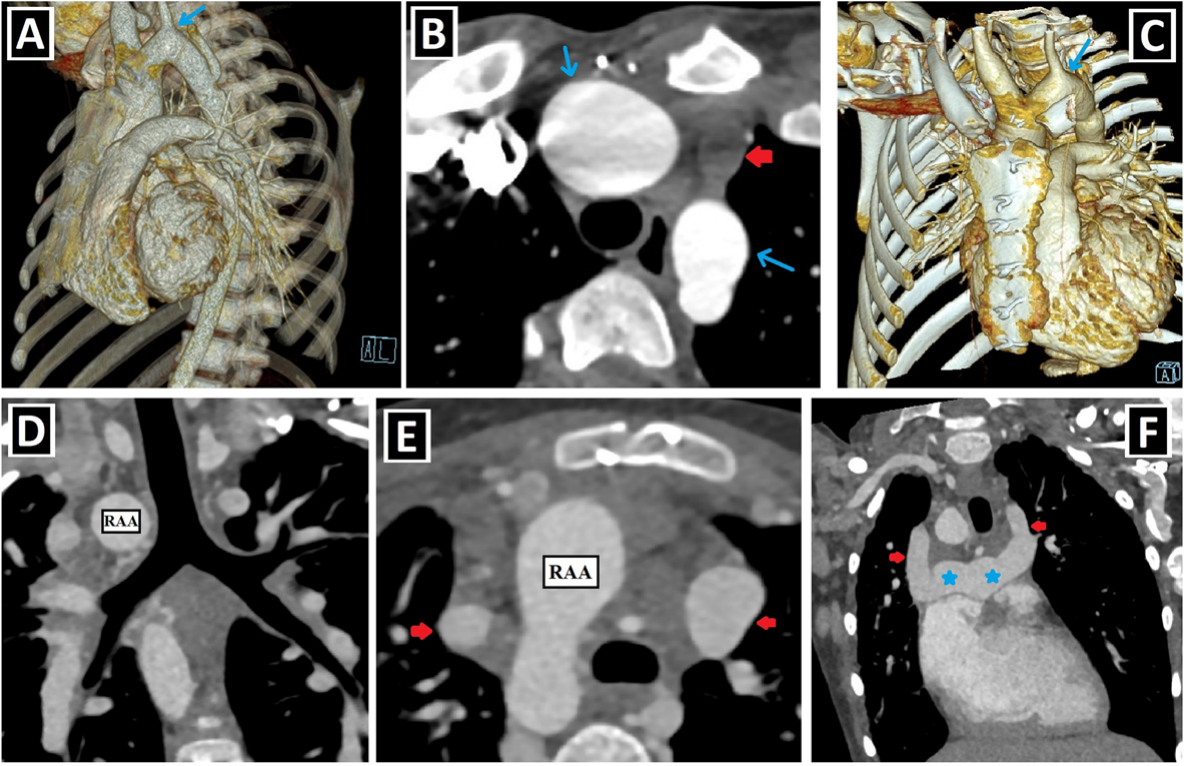
Aortic arch anomalies accompanying PLSVC-2. **a**–**c** 3D VRT images with different views (**a**, **c**), and axial CT image (**b**) indicate the cervical aortic arch (blue arrows) in a patient with PLSVC (red arrows). **d**–**f** Coronal-oblique reformatted (**d**, **f**) and axial (**e**) CT images depict right aortic arch (RAA) and bicaval Glenn shunts (red arrows) in a patient with double SVC and complex cardiac anomaly. Blue stars represent bilateral pulmonary arteries

To date, many CA associated with PLSVC have been identified and grouped in different ways [1, 14, 15]. Shunt lesions (Figs. 4 and 5), conotruncal malformations (CTMs) (Figs. 6 and 7), left-sided obstructive lesions (LOLs) (Fig. 8), right-sided lesions, and single ventricular anomalies (Fig. 9) constitute the main CA groups. Aortic arch anomalies are also associated with PLSVC (Figs. 10 and 11). The subgroups of these anomalies are listed in Table 3. Besides, a summary of the literature about PLSVC and accompanying CAs is compiled in Table 4. Additionally, heterotaxy forms another disease spectrum associated with PLSVC and will be discussed under a separate title.

**Table 3:** Main groups and subgroups of cardiac/aortic arch anomalies associated with PLSVC

Main groups	Subgroups
Shunt lesions	ASD, VSD, AVSD, PDA, APVD
Conotruncal malformations	TOF, PA with VSD, L/D-TGA, TA, DORV
Left-sided obstructive lesions	CoA, cor triatriatum, mitral stenosis, bicuspid aortic valve
Right-sided lesions	PS, PA, tricuspid atresia, bicuspid pulmonary valve, Ebstein anomaly
Single ventricular anomalies	None
Aortic arch anomalies	Cervical arch, RAA, ARSA, RAA + ALSA

*PLSVC* persistent left superior vena cava, *ASD* atrial septal defect, *VSD* ventricular septal defect, *AVSD* atrioventricular septal defect, *PDA* patent ductus arteriosus, *APVD* anomalous pulmonary venous drainage, *TOF* tetralogy of fallot, *PA* pulmonary atresia, *L*/*D* TGA-levo/dextro-transposition of the great arteries, *TA* truncus arteriosus, *DORV* double outlet right ventricle, *CoA* coarctation of the aorta, *PS* pulmonary stenosis, *RAA* right aortic arch, *ARSA* aberrant right subclavian artery, *ALSA* aberrant left subclavian artery

**Table 4:** Summary of literature about PLSVC and accompanying CA

Name of the author	Reported findings of PLSVC and associated CA	
Perles et al. [1]	The most common groups of anomalies associated with PLSVC (Based on odds ratio)	AVSD, CTMs, LOLs
Nagasawa et al. [6]	The highest incidence group of cardiac anomalies according to PLSVC index	CoA and DORV
Lendzidan et al. [14]	The most common cardiac anomalies associated with PLSVC	Single ventricle, AVSD, TOF
Ari et al. [12]	The most common cyanotic heart diseases associated with PLSVC	DORV and TOF
	The most common acyanotic heart diseases associated with PLSVC	ASD and PDA
Berg et al. [13]	The most common concomitant anomalies in patients with heterotaxy and PLSVC	AVSD, RVOTO, DORV
	The most common concomitant anomalies in patients with PLSVC, without heterotaxy	VSD and CoA
Oztunc et al. [16]	The most common anomalies with PLSVC drained into the right atrium	TOF and PS
	The most common anomalies with PLSVC drained into the left atrium	Tricuspid atresia, TGA, situs anomalies

*PLSVC* persistent left superior vena cava, *CA* cardiac anomaly, *AVSD* atrioventricular septal defect, *CTMs* conotruncal malformations, *LOLs* left-sided obstructive lesions, *CoA* coarctation of the aorta, *DORV* double outlet right ventricle, *TOF* tetralogy of fallot, *ASD* atrial septal defect, *PDA* patent ductus arteriosus, *RVOTO* right ventricular outflow tract obstruction, *VSD* ventricular septal defect, *PS* pulmonary stenosis, *TGA* transposition of great arteries

In the literature, there is a wide range of information about the frequency of cardiac anomalies accompanying PLSVC [1, 6, 14–22]. According to Lendzidan et al., the most common cardiac anomalies accompanying PLSVC are single ventricle, atrioventricular septal defect (AVSD), and tetralogy of Fallot (TOF). Cha et al. reported that the most frequent concomitant anomaly is ASD, whereas, according to Eldin et al., complete atrioventricular septal defect comes the first [17–19].

Moreover, attention has been drawn to the relationship of some specific cardiac anomalies with PLSVC in many publications. In addition to left-sided pathologies such as mitral atresia, cor triatriatum, and hypoplastic left heart, transposition of the great arteries (TGA) and tricuspid atresia are other rarer anomalies that have been reported to be closely related to PLSVC in the literature [6, 16, 20].

Different cardiac anomalies come to the fore in different situations such as type of accompanying cardiac anomaly (cyanotic or acyanotic), presence of heterotaxy, and drainage location of PLSVC [14, 15, 21]. Different parameters, such as odds ratio and PLSVC index, are calculated in the literature and used to determine the relationship between PLSVC and cardiac anomaly [1, 8]. In some publications, cardiac anomalies accompanying PLSVC were grouped and evaluated as in Table 3, and in others, they were examined separately [1, 17].

Association of PLSVC with aorta-related pathologies such as right-sided arcus aorta (RAA) and coarctation of the aorta (CoA) have also been emphasized in the literature. It was mentioned that the association of PLSVC with RAA is approximately 16% [14]. In another study, CoA was reported to be an independent and powerful factor for the existence of PLSVC [8]. Gustapane et al. underlined the coexistence of PLSVC with coarctation of the aorta (CoA) (21.3%) and suggested that fetuses with PLSVC that are detected in the antenatal period should be followed during pregnancy in terms of CoA development [22].

## PLSVC and heterotaxy

**Fig. 12:**
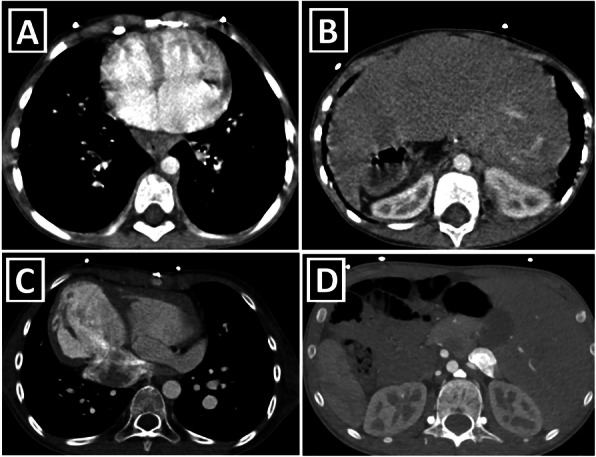
PLSVC with heterotaxy-1 (situs ambiguus and situs inversus). **a**, **b** Axial CT images depict mesocardia (**a**), midline liver (**b**), right-sided stomach (**b**), and absence of spleen (**b**) in a patient with situs ambiguus and PLSVC (not shown). **c**, **d** Axial CT images show situs inversus characterized by dextrocardia (**c**), right-sided stomach and spleen (**d**), and left-sided liver (**d**) in a patient with double SVC (not shown)

The term heterotaxy comprises situs inversus and situs ambiguus (right/left isomerism) (Fig. 12). DSVC or IPLSVC anomalies may be present in patients with heterotaxy. Meanwhile, in a study, “patients with both IPLSVC and situs inversus” were considered normal because of mirror image and excluded. In contrast, “patients with both isolated RSVC and situs inversus” were regarded as abnormal and accepted as SVC anomaly [1].

According to the literature, PLSVC-heterotaxy coexistence is frequently observed, and PLSVC is present in 50–70% of heterotaxy cases. Additionally, it is informed that 45% of patients with PLSVC in the antenatal period have accompanying heterotaxy [15]. In a study, DSVC is detected in nearly half of patients with heterotaxy [8]. Another study reported that 72% of heterotaxy patients with SVC anomaly have DSVC, while the remaining have IPLSVC [23].

**Fig. 13:**
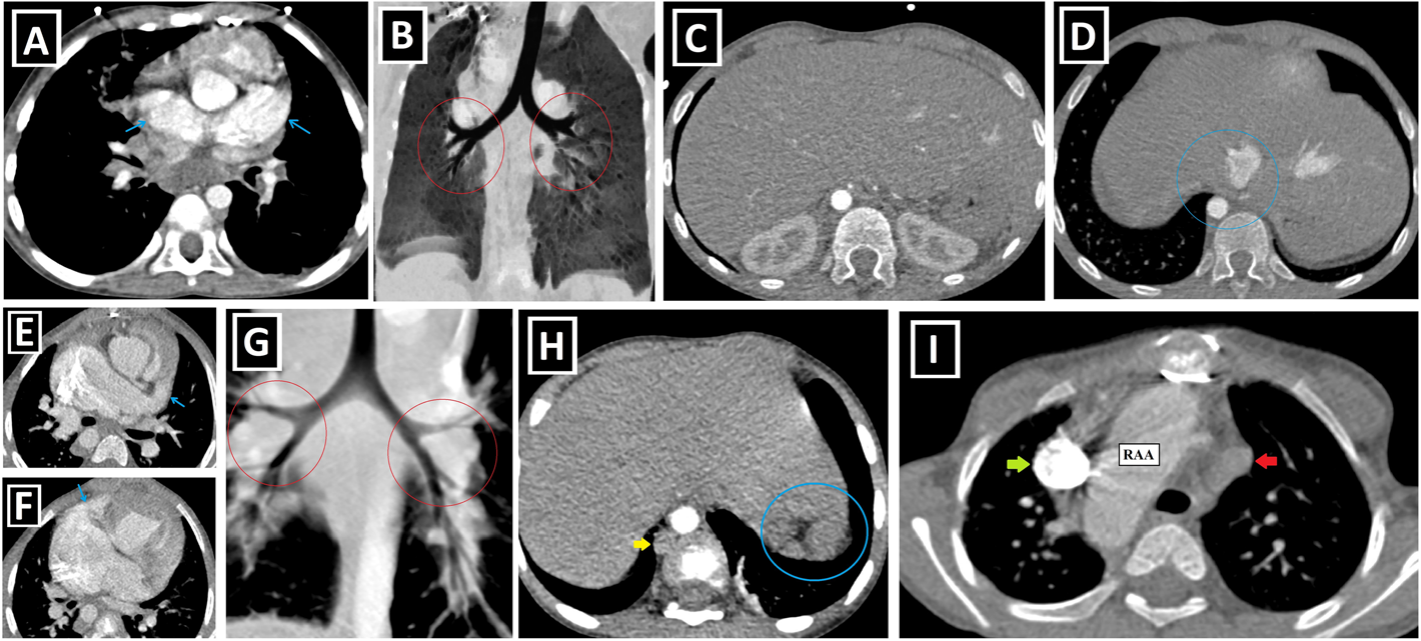
PLSVC with heterotaxy-2 (right and left isomerisms). **a**–**d** Axial (**a**, **c**, **d**) and coronal-oblique minimum intensity projection (**b**) CT images depict right isomerism characterized by a bilateral broad-based triangular atrial appendages (blue arrows) (**a**), bilateral trilobed lungs (red circles) (**b**), asplenia (**c**), and juxtaposition of IVC (blue circle) in a patient with PLSVC (not shown). **e**–**i** Axial (**e**, **f**, **h**, **i**) and coronal-oblique reformatted (**g**) CT images depict left isomerism characterized by a bilateral narrow-based finger-like atrial appendages (blue arrows) (**e**, **f**), bilateral bilobed lungs (red circles) (**g**), polysplenia (blue circle) (**h**), dilated azygos vein due to the IVC absence (yellow arrow) (**h**), and right aortic arch (RAA) in a patient with double SVC (green and red arrows) (**i**)

According to a study, while right atrial isomerism in patients with PLSVC is about 7%, left atrial isomerism is about 9% [14]. In another study, those prevalences are nearly 15% and 30%, respectively. It was stated that the absence of inferior vena cava (IVC) is associated with left atrial isomerism, while the juxtaposition of IVC is observed in right atrial isomerism [15] (Fig. 13).

**Fig. 14:**
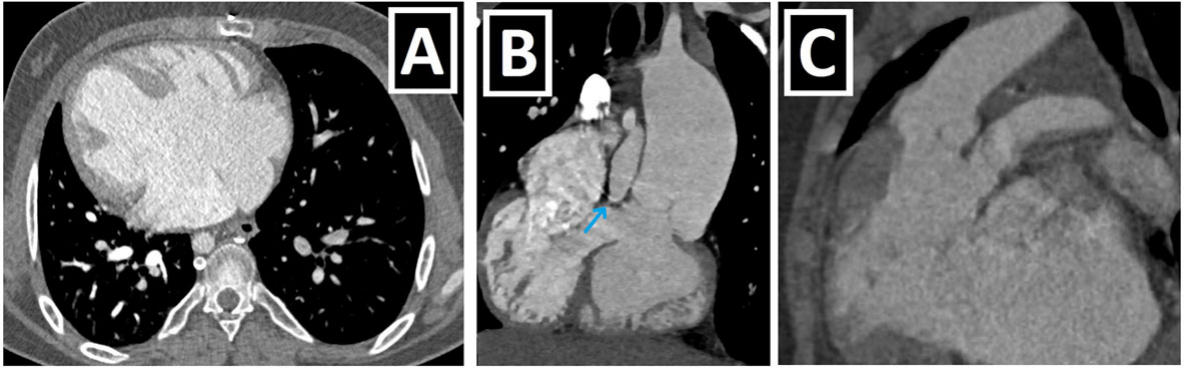
PLSVC with heterotaxy-3 (the most common accompanying cardiac anomalies). Axial (**a**), coronal-oblique (**b**), and sagittal-oblique reformatted (**c**) CT images depict the three most common cardiac anomaly seen in the PLSVC and heterotaxy coexistence: AVSD (with dextrocardia) (**a**), pulmonary atresia (with VSD) (blue arrow, **b**) and DORV (**c**)

Complete atrioventricular septal defect, right ventricular outflow tract obstruction (RVOTO) (pulmonary stenosis and atresia), and double outlet right ventricle (DORV) are found as the most common accompanying anomalies of PLSVC in patients with heterotaxy [15] (Table 4, Fig. 14). The presence of concomitant heterotaxy and atrioventricular septal defect in patients with PLSVC during the antenatal period has been associated with poor prognosis.

Berg et al. reported that they never saw CS dilatation, which is a well-known sonographic finding supporting the presence of PLSVC, in the heterotaxy group. The absence of CS dilatation has been associated with unroofed CS, which is found in almost all heterotaxy cases. It should be kept in mind that the absence of CS dilatation does not exclude the presence of PLSVC in patients with heterotaxy during the antenatal period, and the possibility of concomitant unroofed CS anomaly is high [15].

## Clinical importance

The clinical significance of PLSVC depends on the drainage site and the accompanying anomalies. PLSVC without CA is generally asymptomatic and is detected as an incidental finding. In the case of PLSVC with right atrial drainage, the CS often expands (Fig. 2g, h). This enlargement may cause compression of the atrioventricular node and His bundle. So, it can lead to cardiac arrhythmias, such as atrial/ventricular fibrillation. The compression of the left atrium and decreased cardiac output may occur due to this enlargement. Moreover, the presence of CS dilatation may complicate mitral valve surgery due to the close anatomic relationship [2, 9, 12, 19].

In a recent study by Yun Gi Kim et al. [24], it was demonstrated that PLSVC plays a considerable role in the induction and maintenance of atrial fibrillation (AF) in nearly half of the patients. So, pre-radiofrequency catheter ablation cardiac imaging in AF patients is useful and necessary for not only the evaluation of pulmonary venous anatomy but also for the detection of PLSVC existence. If PLSVC is detected as the trigger or driver of AF, it can be ablated (Fig. 2c).

It is crucial to know the PLSVC existence in advance in invasive procedures, such as central venous catheter (CVC) insertion (Fig. 2a, b), cardiac resynchronization therapy leads, or pacemaker implantation. It may complicate pacemaker implantation by causing fixation difficulties of the electrode due to the tortuous course. CVC insertion without fluoroscopy may cause angina, hypotension, and heart perforation. Furthermore, there may be constriction or atresia of the CS ostium. In this case, the catheterization will be challenging and may result in serious complications, such as dangerous arrhythmias, cardiogenic shock, and tamponade [2, 9, 12, 14].

The presence of CS ostial atresia is also critical in the operations that require PLSVC ligation. In this case, the CS still drains the blood from the coronary veins to the right atrium via the retrograde PLSVC-LBCV-RSVC pathway, instead of the atretic ostium. The ligation of PLSVC will be catastrophic due to the acute interruption of the cardiac venous drainage [12].

The left atrial drainage of PLSVC (Fig. 2d–f), sometimes, remains asymptomatic because it does not cause a right-to-left shunt at a significant level. In cases where the shunt is more pronounced, as a result of desaturation, the condition manifests itself with severe cyanosis, syncope, reduced exercise tolerance, and progressive fatigue. Thromboembolic events and even brain abscesses may develop in these patients. In this case, treatment can be done in two ways based on anatomy: PLSVC can be ligated if there is an adequate sized BV, and PLSVC can be re-anastomosed to the CS if the BV is not adequate in size or there is no RSVC [2, 9].

The knowledge of PLSVC is fundamental in some cardiac surgeries such as venous rerouting procedures, operations with cavo-pulmonary anastomosis (Glenn, Fontan), and heart transplantation. In heart transplantation surgery, if PLSVC without BV is present in the recipient’s heart, the bicaval anastomosis technique will be performed. It requires separation of the CS of the donor’s heart for the establishment of the recipient’s PLSVC anastomosis to the donor’s right atrium [1].

In the case of unknown PLSVC, retrograde cardioplegia, a common practice for cardiac surgeries for myocardial protection, will be ineffective. Clamping of PLSVC may be required for the prevention of retrograde flow. However, cardioplegia may fail even after clamping of PLSVC, due to the steal effect by the hemiazygos venous system linked to PLSVC [1, 3].

During cardiopulmonary bypass, not knowing PLSVC existence may result in both surplus blood return through the right atrium and insufficient venous return to the pump. This problem is mostly encountered in pathologies such as pulmonary atresia, tricuspid atresia, TOF, where increased systemic venous pressure gets over the level of left atrial pressure [19].

With the help of screening echocardiography, PLSVC can be detected as early as in the prenatal period. It can be used as a marker for cardiac or non-cardiac embryopathy. It may require extensive evaluation to exclude possible developmental anomalies. In cases with CHD, symptoms will be mainly due to these anomalies [1].

## Pitfalls and differential diagnoses

In the presence of the vessel on the left side of the aorta in the mediastinum, other vascular structures apart from PLSVC should be considered in the differential diagnosis. They are vertical vein, levoatriocardinal vein, left superior intercostal vein, aberrant left brachiocephalic vein, pericardiophrenic vein, and vascular structures secondary to surgery.

**Fig. 15:**
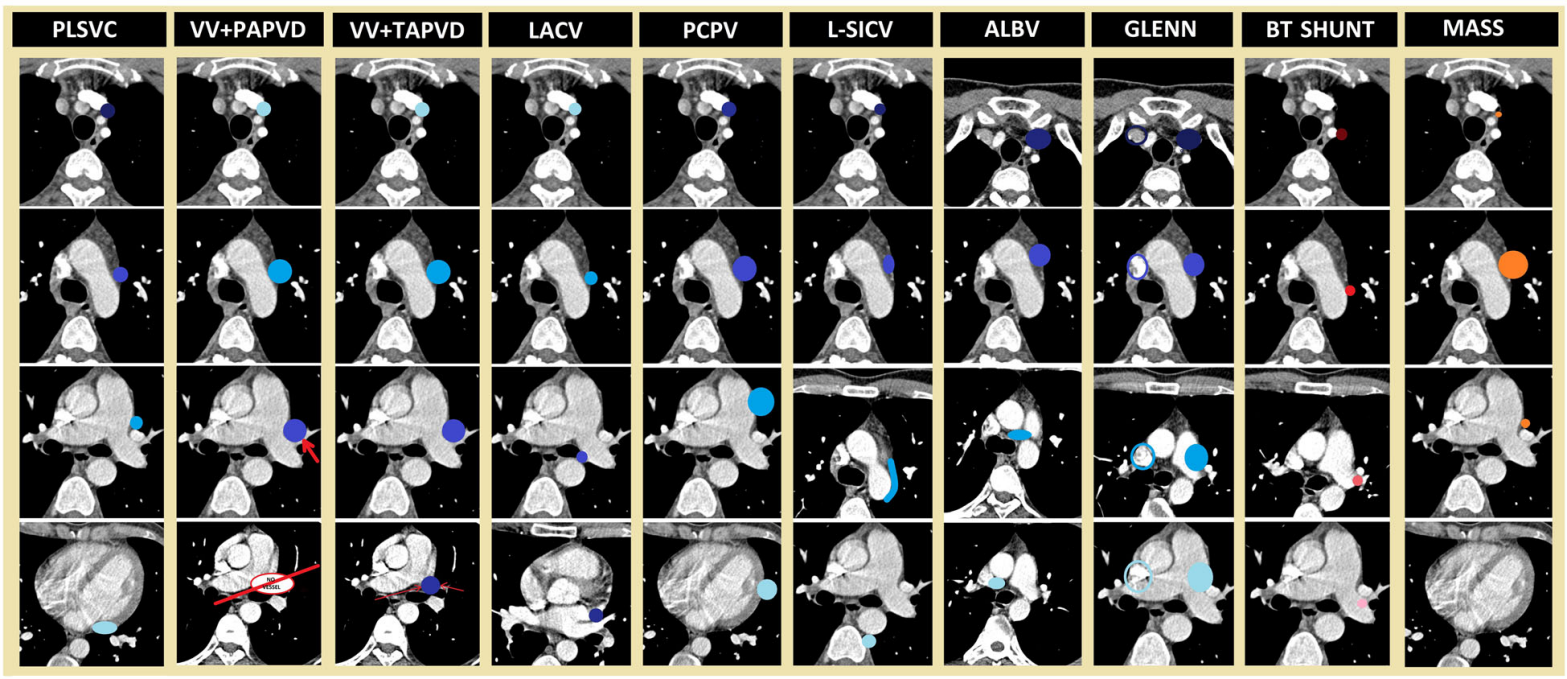
A comprehensive summary of differential diagnoses of PLSVC. The course of the most frequent PLSVC variation (right atrial drainage via coronary sinus) and the courses of possible differential diagnoses are shown as columns with axial CT images from superior to inferior. For depicting the expected flow direction of vascular structures, the upstream zones are marked with darker shades, whereas the downstream zones with lighter shades. Red arrows represent pulmonary veins. Blue circles are used to depict systemic venous structures, while red circles are used to show the BT shunt, which is an interarterial structure. Orange circles represent the mass. In the column of Glenn shunt, the course of PLSVC is shown with solid blue circles while the course of RSVC is shown with hollow blue circles. Please note that (1) in the second row of the figure, all differential diagnoses of PLSVC are observed to be in a similar location in the mediastinum. (2) While two vascular structures are seen in front of the left main bronchus in the presence of PLSVC, no vascular structure is seen in this area in the presence of VV with PAPVD. (3) Expected flow directions fo VV and LACV are caudocranial, unlike other vascular structures shown in the figure. Also, LACV is located in the posterior of the pulmonary artery, unlike PLSVC, which is located anterior to the pulmonary artery

To make the definitive diagnosis, features which should be taken into consideration are as follows: “origin site,” “drainage site,” “orientation of the route between the origin and drainage site according to mediastinal structures,” “the expected direction of the blood flow,” and “characteristics of accompanying cardiac and non-cardiac diseases.” According to the above-mentioned features, a comprehensive summary of the differential diagnoses of PLSVC is depicted in Fig. 15.

**Fig. 16:**
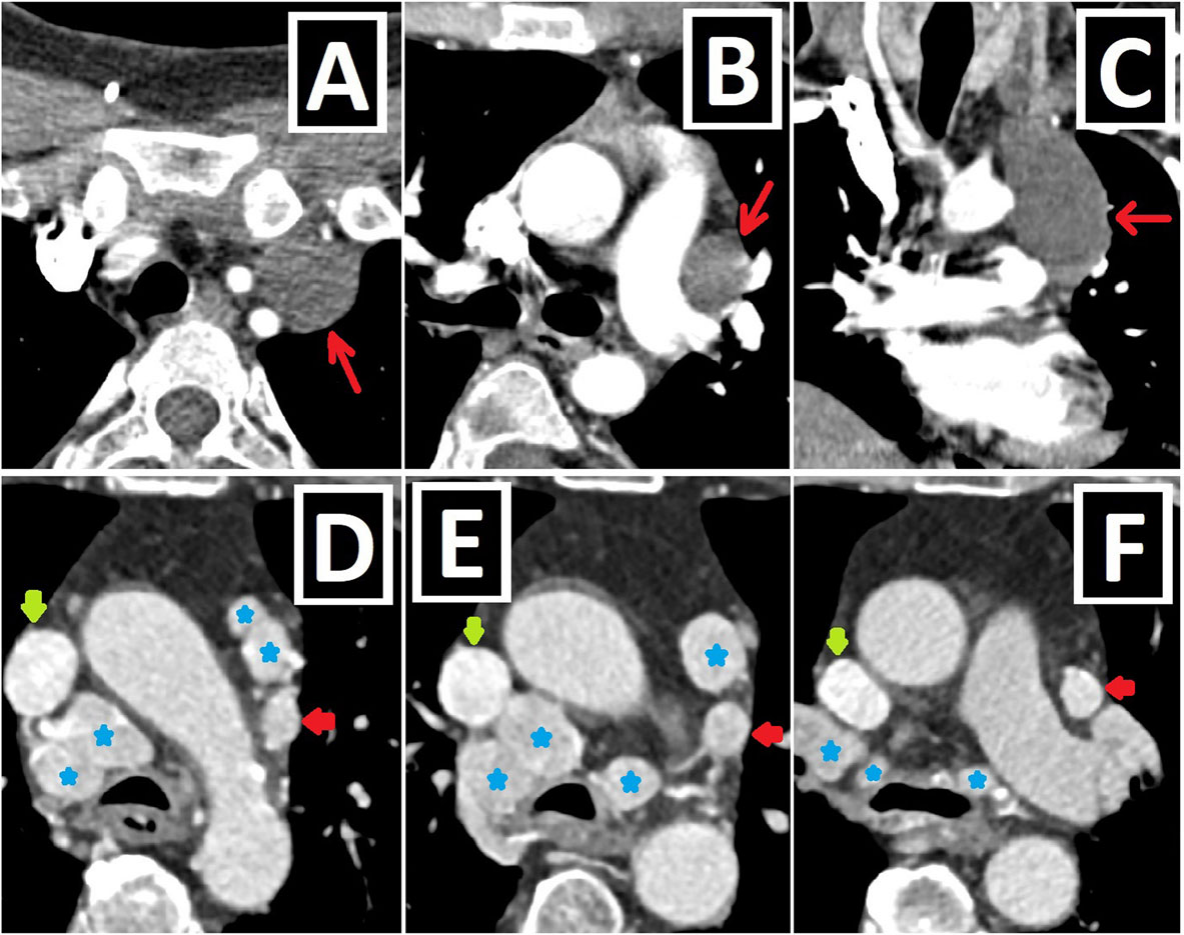
Masses mimicking PLSVC. **a**–**c** Axial (**a**, **b**) and coronal-oblique reformated (**c**) CT images depict a neurofibroma (red arrows) arising from the left phrenic nerve, which has a parallel course with the pericardiophrenic vein, in a patient with neurofibromatosis type 1. If the beginning/end of the mass and relationship with vascular structures are not carefully evaluated, it can be confused with PLSVC due to its location. **d**–**f** Axial CT images depict multiple mediastinal hypervascular lymphadenopathies (blue stars) in the patient with renal cell carcinoma. Hypervascular lymphadenopathies in the left half of the mediastinum (red arrows) may mimic PLSVC. Green arrows show RSVC

Some masses on the expected course of PLSVC could be confusing at first look due to their location. For making the differential diagnosis, it is essential to follow all of the slices carefully and see the beginning and end of the mass (Fig. 16) [25, 26].

Moreover, an interesting variant of PLSVC, which has an intra-atrial course within the left atrium, has been identified recently. If this pitfall variant is not known, it may be misunderstood as left atrial cystic mass, may cause patient anxiety, and may lead to unnecessary effort for further investigations [27].

### Vertical vein

**Fig. 17:**
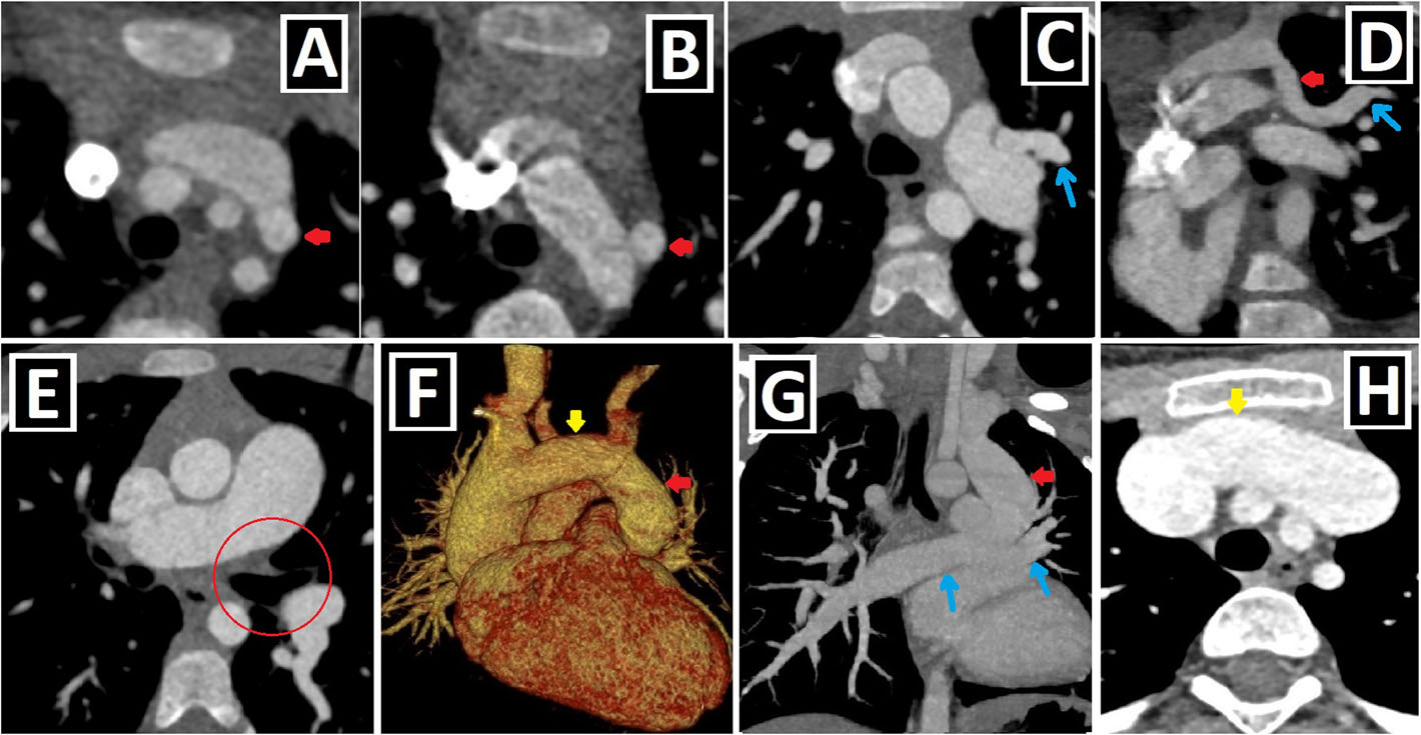
Vertical vein. **a**–**e** Axial (**a**, **b**, **c**, **e**) and coronal-oblique reformated (**d**) CT images depict abnormal drainage of the left upper pulmonary vein (blue arrows) into the left brachiocephalic vein via the VV (red arrows). In the presence of VV with PAPVD (left-upper), the absence of vascular structure anterior to the left main bronchus (red circle) is an important clue for differential diagnosis (**e**). **f**–**h** 3D VRT (**f**), coronal-oblique maximum intensity projection (**g**), and axial (**h**) CT images depict the drainage of both right and left pulmonary veins (blue arrows) into the VV (red arrows), in a patient with supracardiac type TAPVD. The VV transports the whole pulmonary venous blood to the SVC through a large-caliber bridging vein (yellow arrows)

The vertical vein (VV) is the vessel that drains the blood from the pulmonary veins into the LBCV in the presence of supracardiac type total or partial APVD (TAPVD or PAPVD) (Fig. 17). It may be left- or right-sided. The left APVD accounts for approximately 18% of all PAPVD and left superior pulmonary veins are affected mostly. The left-sided VV is one of the differential diagnoses of PLSVC. The critical point in the distinction is the caudal continuity of the vessel with atrial chambers. If there is no continuity, it is compatible with the VV. However, PLSVC may have a direct connection with the left pulmonary veins. In this scenario, the pulmonary vein drains into the left atrium after joining PLSVC [12, 28].

There are also some auxiliary features to differentiate PLSVC and VV. The expected flow direction is craniocaudal in PLSVC, while it is caudocranial in the VV. In the case of PLSVC, there are two vessels in the anterior aspect of the left main bronchus: one of them is PLSVC, and the other one is the left superior pulmonary vein. Ordinarily, only the left superior pulmonary vein is expected to be at this location. However, in the case of the VV with PAPVD, no vessel is seen in the anterior aspect of the left main bronchus. The size of the LBCV can also be helpful in finding for differentiation. In the case of APVD, the LBCV and RSVC may be of large caliber because the VV transports blood via these venous structures. On the other hand, PLSVC, frequently, is associated with an absent or small-sized LBCV [12, 28].

### Levoatriocardinal vein

**Fig. 18:**
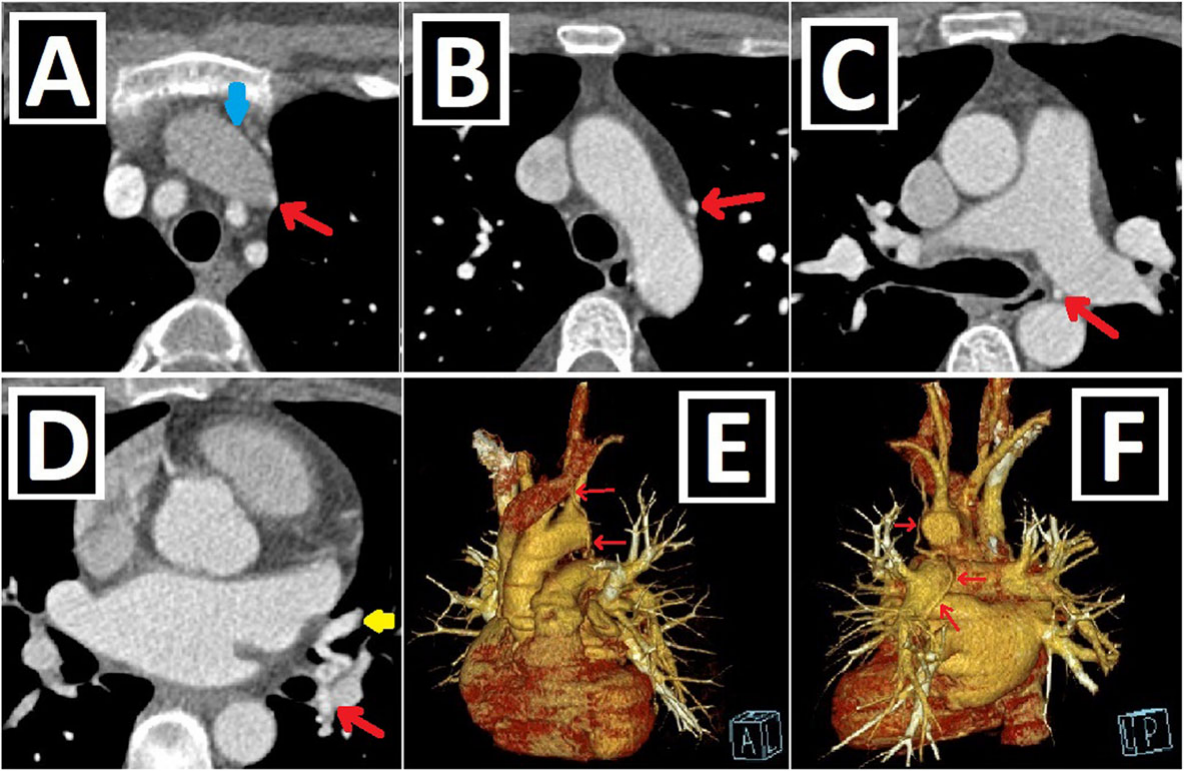
Levoatriocardinal vein-1. **a**–**f** CT images of a patient with mitral valve stenosis due to acute rheumatic fever. The first four axial CT images (**a**–**d**) depict the venous structure (red arrows) coursing between the left brachiocephalic vein (blue arrow) and the left upper pulmonary vein (yellow arrow). The density difference between the cranial and caudal ends of the vessel suggests that flow direction is caudo-cranial. 3D VRT CT images with anterior and posterior views (**e**, **f**) show the course of the vessel. This vascular structure observed in the posterior of the pulmonary artery in the patient with mitral stenosis, which is a LOL, is compatible with LACV (red arrows)

**Fig. 19:**
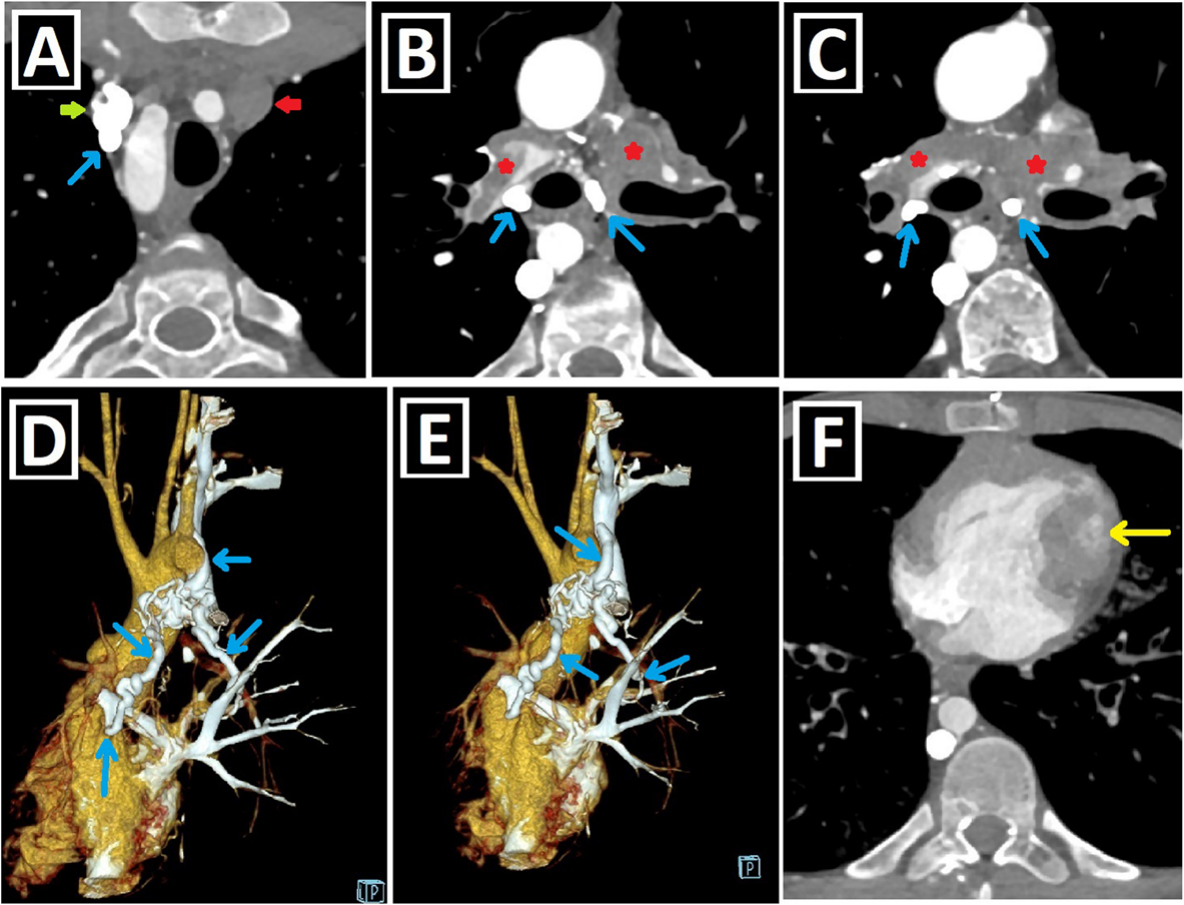
Levoatriocardinal vein-2. Axial (**a**, **b**, **c**, **f**) and 3D VRT (**d**, **e**) CT images of a patient with double SVC (green and red arrows, **a**) and complex cardiac anomaly, who underwent bicaval Glenn shunt operation. Axial CT images (**b**, **c**) depict the thrombus extending from the right Glenn shunt to confluent pulmonary arteries (red stars). Due to the thrombus in the right Glenn shunt, the distribution of the contrast agent injected from the right arm into the mediastinal collaterals and the azygos system is observed. Axial (**b**, **c**) and 3D VRT reconstructed (**d**, **e**) CT images indicate bilateral vascular structures, which are compatible with LACV (blue arrows), originating from right Glenn shunt, coursing in the posterior of bilateral pulmonary arteries and draining into the right and left upper pulmonary veins. Please note that LACV may accompany LOLs (yellow arrow shows hypoplastic left heart, **f**), may be seen together with PLSVC, may be associated with any venous structure in the cardinal system not only left brachiocephalic vein, and in some cases, may have blood flow in the craniocaudal direction

The levoatriocardinal vein (LACV) is the interatrial connection that originates from the left atrium (68%) or pulmonary vein (32%). It drains into one of the systemic venous structures, mostly, into the LBCV (48%) (Figs. 18 and 19) [29, 30].

The differentiation of LACV from PLSVC with right atrial drainage is straightforward. Because this drainage site is unlikely for the levoatriocardinal vein. Similarly, in cases where PLSVC drains into the left atrium via the unroofed CS, unroofed CS and ASD facilitate the differential diagnosis in favor of PLSVC, since they are unusual for LACV [29–31].

However, PLSVC may drain directly into the left atrium or pulmonary vein. In this situation, the expected origin and drainage site of those two vessels will be the same, and it is necessary to search other features for distinguishment. The anatomical feature that may help distinguish is their relative orientation according to the left pulmonary artery. PLSVC is seen in the anterior aspect of the left pulmonary artery, while the LACV is in the posterior aspect. The evaluation of the flow direction with echocardiography or velocity-encoded cine magnetic resonance imaging is another way to make differential diagnoses. The blood flows in the caudocranial direction in the LACV while it flows craniocaudal direction in the PLSVC. However, the bidirectional flow could be seen in the levoatriocardinal vein [29–31].

Moreover, the caudocranial flow may be observed in PLSVC when there is atresia or stenosis of the CS ostium. Identification of accompanying CA may also help in the differential diagnosis. If LOLs without ASD are present, LACV should be considered in the differential diagnosis, firstly. It is hypothesized that, in the presence of in utero LOLs such as mitral stenosis, collaterals between pulmonary and systemic circulations cannot regress due to increased pressure in the left atrium and remain as LACV in the postnatal period. However, in the presence of complex CA, the diagnosis of PLSVC should be considered mainly [29–31].

LACV could be isolated without any CA, like PLSVC. Nevertheless, the frequency of this probability is very low for LACV compared to PLSVC. Additionally, they may be seen together, and the LACV may drain into PLSVC [28, 30].

### Pericardiophrenic vein

**Fig. 20:**
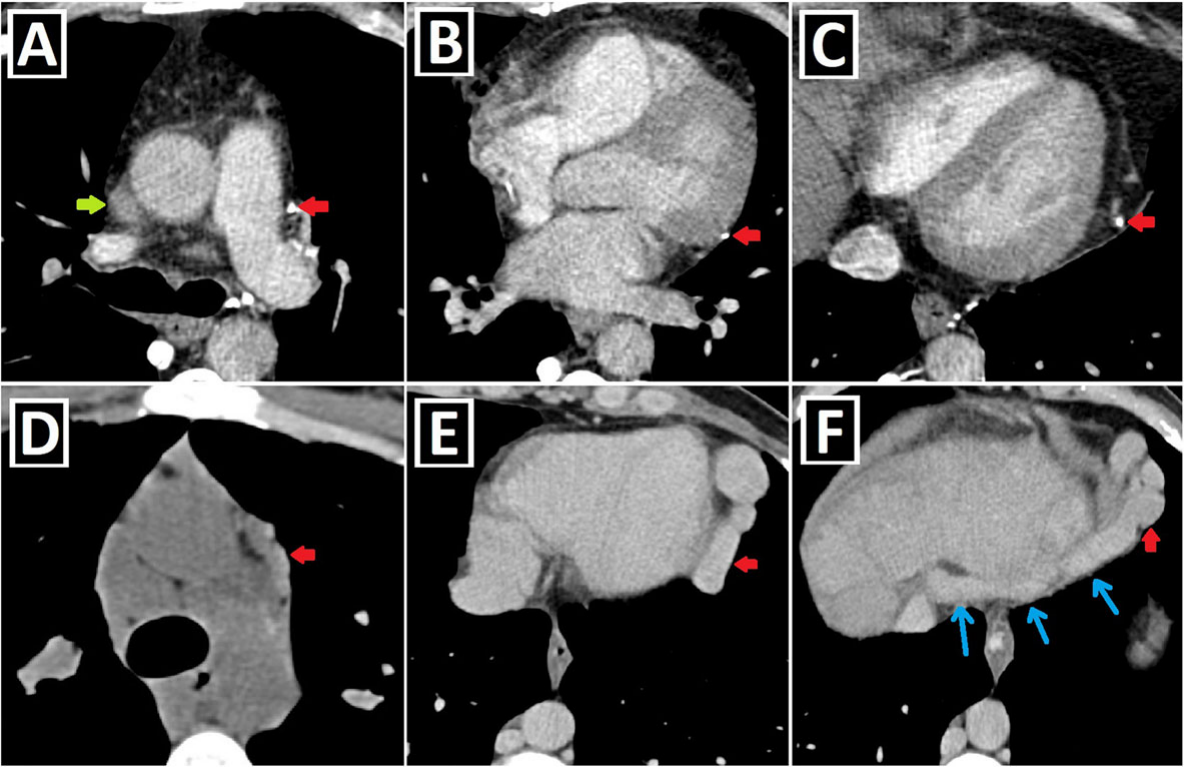
Left pericardiophrenic vein. **a**–**c** Axial CT images show a vascular structure compatible with PCPV (red arrows) in the left half of the mediastinum in a patient with SVC stenosis (green arrow). **d**–**f** Axial non-enhanced (**d**) and enhanced (**e**, **f**) CT images of a patient with portal hypertension secondary to Budd-Chiari syndrome depict varicose veins compatible with PCPV (red arrows) in the left half of the mediastinum. These vascular structures are connected with hepatic veins via a transdiaphragmatic course (blue arrows) (**f**)

The pericardiophrenic veins (PCPV) are responsible for pericardial and diaphragmatic venous drainage. They lie along the lateral border of the heart and mediastinum, accompany pericardiophrenic arteries/phrenic nerve and drain into the internal thoracic, superior intercostal, or BCV. Due to the connection with inferior phrenic veins, dilated PCPVs could be observed as a collateral pathway in cases of SVC or IVC occlusion. Besides, they can serve as a collateral route via portosystemic shunting in portal hypertension (Fig. 20) [32–34].

In the case of catheters located at the left paramediastinal region, the left PCPV is one of the possible differential diagnoses. In posteroanterior chest X-ray, left PCPV has a lateral course along the left heart border, while PLSVC turns medially near the left atrium. Although they both are located in the middle mediastinum and connected with left brachiocephalic vein cranially, their caudal courses differ in CT imaging. While the caudal end of PLSVC is either the coronary sinus or the left atrium, the left pericardiophrenic vein moves toward the diaphragm lateral to the heart when it is followed from top to bottom [35, 36].

### Left superior intercostal vein

**Fig. 21:**
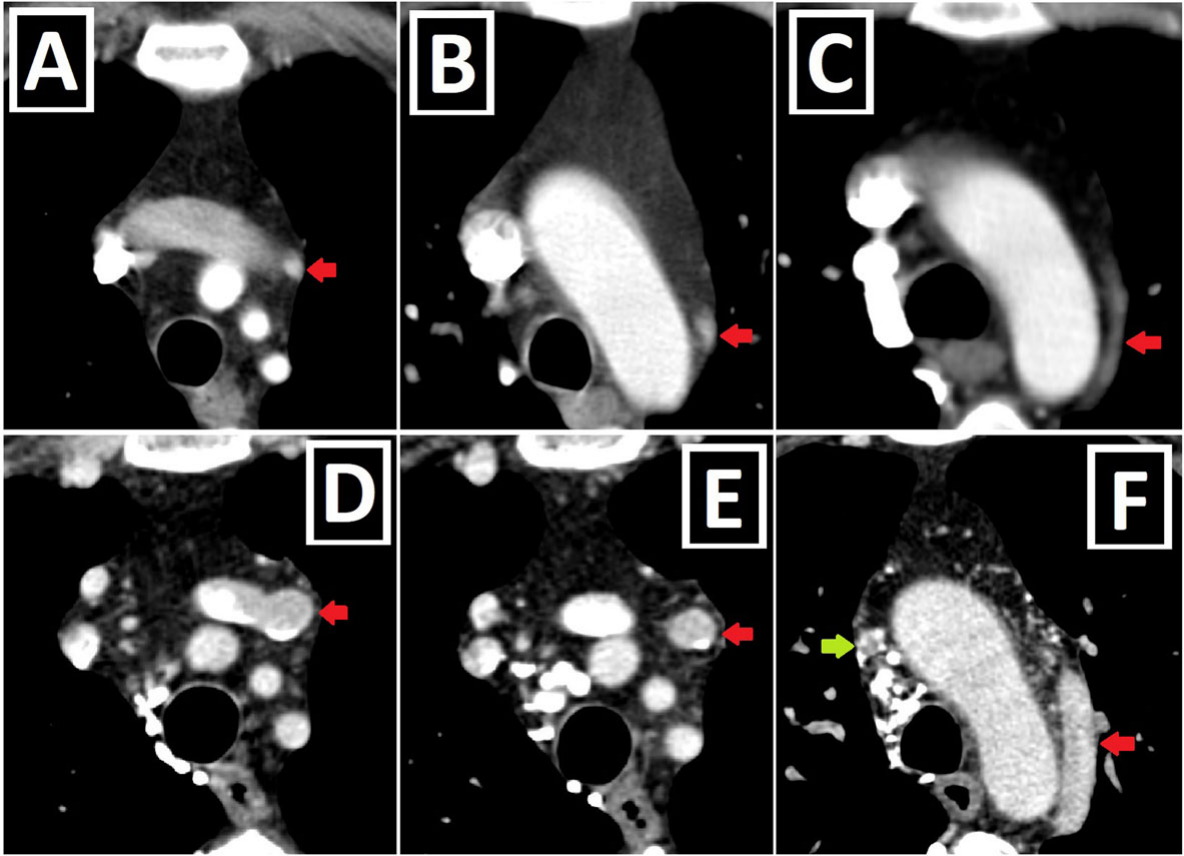
Left superior intercostal vein. **a**–**c** Axial CT images depict a thin-caliber LSIV (red arrows) in a healthy patient. **d**–**f** After developing SVC occlusion (green arrow, **f**), axial CT images show multiple mediastinal venous collaterals and markedly enlarged LSIV (red arrows) in the same patient

The left superior intercostal vein (L-SICV) drains the blood from the second, third, and fourth left intercostal veins into RSVC through the hemiazygos/azygos venous systems. Ordinarily, it can be seen as a small aortic nipple (1.4–5%) on the chest radiograph and is indistinguishable in CT. If its diameter exceeds 4.5 mm, it should be considered as abnormal. In the case of occlusion of SVC at the distal level of the azygos vein, the connection between SVC and IVC becomes possible with the dilation of L-SICV and other collateral vessels (Fig. 21) [9, 37, 38].

Furthermore, L-SICV may dilate in congenital conditions such as hypoplasia of LBCV, and diseases leading to volume overload such as congestive heart failure. In such cases, L-SICV might be confused with PLSVC. However, knowing their courses and drainage sites will facilitate the diagnosis [9, 37, 38].

### Aberrant left brachiocephalic vein

**Fig. 22:**
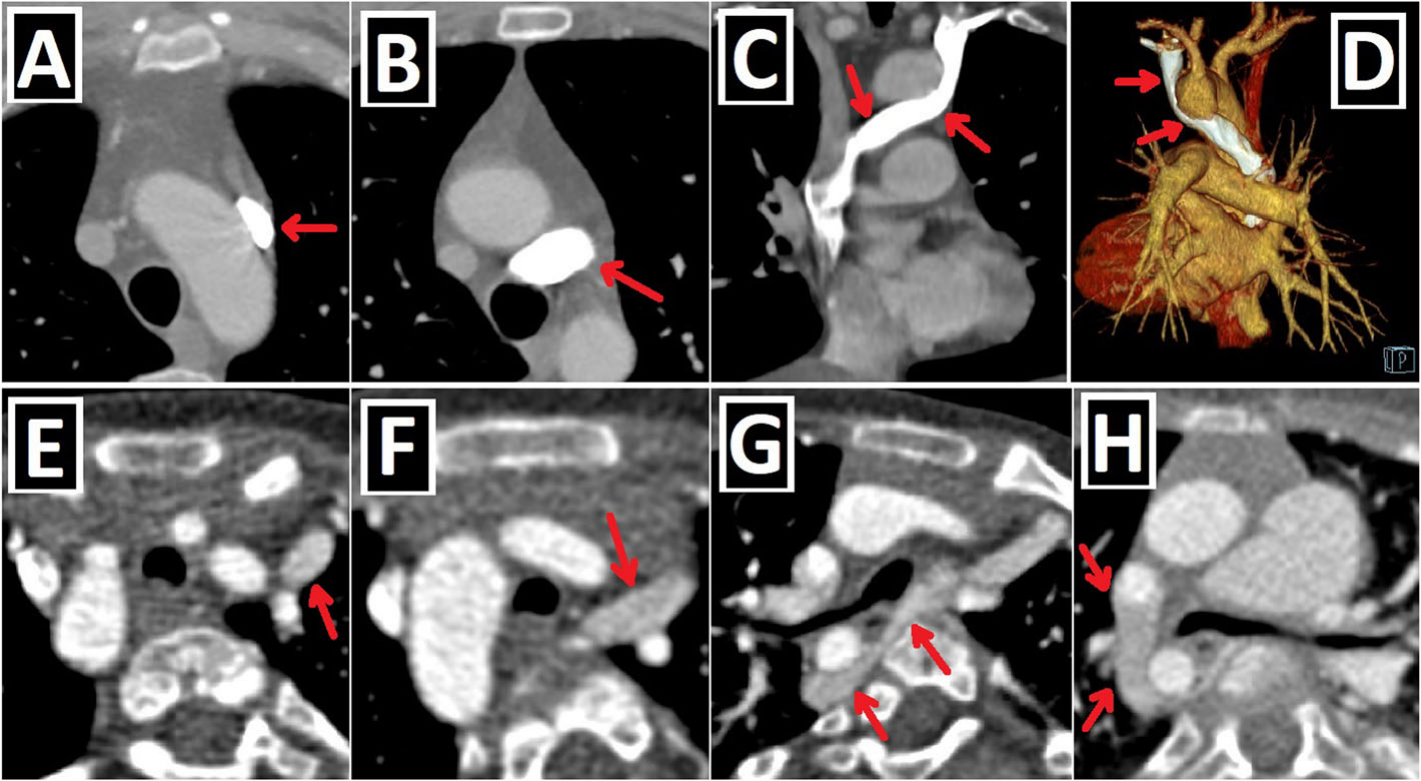
Aberrant left brachiocephalic vein. **a**–**d** Axial (**a**, **b**), coronal-oblique reformatted (**c**), and 3D VRT (**d**) CT images depict the anomalous subaortic course of the left brachiocephalic vein (red arrows). Note that no venous structure is observed in the left half of the mediastinum below the aberrant left brachiocephalic vein level. **e**–**h** Axial CT images depict an aberrant left brachiocephalic vein with an anomalous retroesophageal course (red arrows)

Aberrant left brachiocephalic vein (ALBV) is a rare anomaly (≈ 1%) and is often associated with CAs, such as TOF, septal defects, and right atrial isomerism. Ordinarily, the LBCV passes through the anterior of the arcus aorta and connects with the right BCV. In the presence of an aberrant course, the LBCV begins with the junction of the left subclavian and jugular veins, moves inferiorly along the left side of the mediastinum, and joins to the right BCV passing behind the ascending aorta or esophagus. Retroesophageal ALBV is a more rare variation (Fig. 22) [10, 11, 39].

### Vascular structures secondary to surgery

**Fig. 23:**
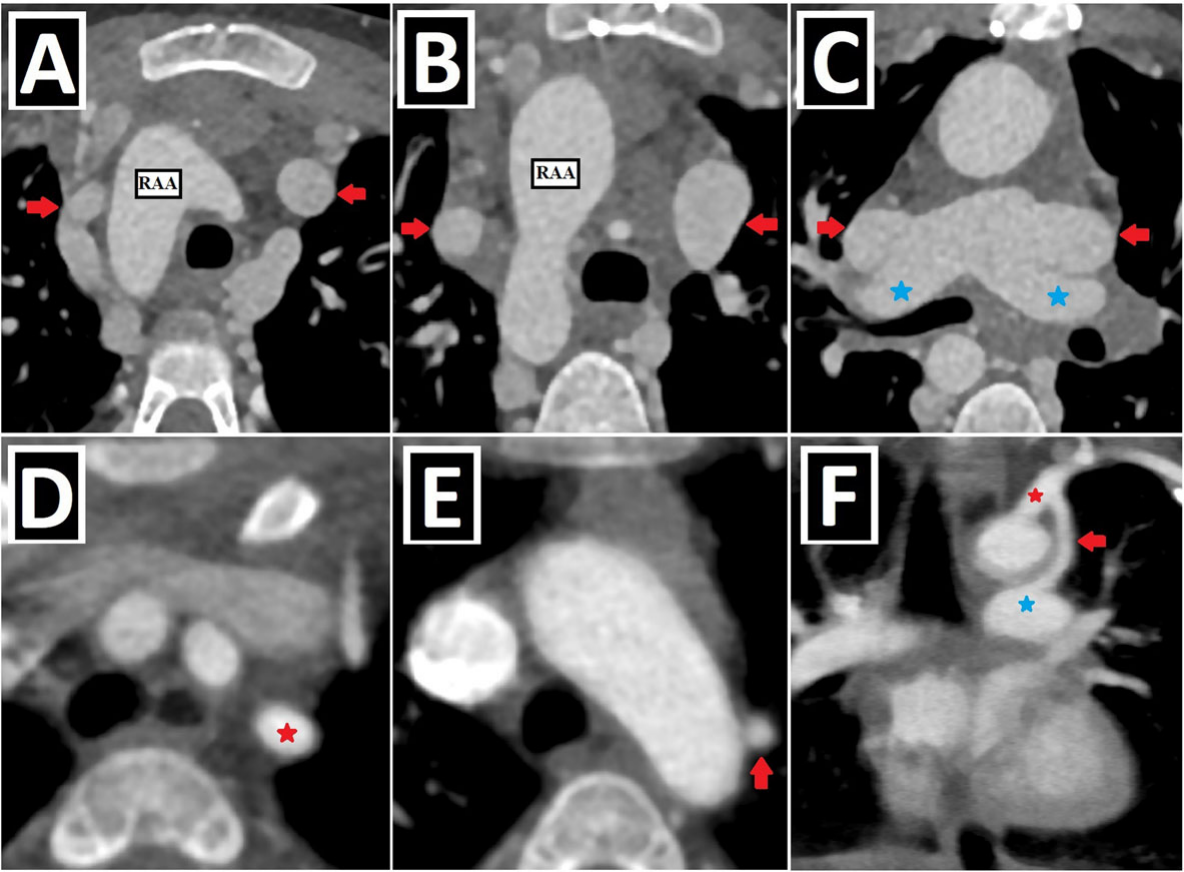
Vascular structures secondary to surgery. **a**–**c** Axial CT images of the patient with the right aortic arch (RAA) and complex cardiac anomaly depict bicaval Glenn shunt (red arrows), which is an anastomosis constructed between the right and left SVCs and right and left pulmonary arteries (blue stars, **c**), respectively. After the performing surgical connection between the PLSVC and the left pulmonary artery, the PLSVC draining to the left pulmonary artery is called the Glenn shunt. **e–f** Axial CT images of the patient with the TOF show BT shunt (red arrows) connecting the left subclavian artery (red stars) and the left pulmonary artery (blue star). The distinction of PLSVC from the BT shunt is quite easy by establishing that the vascular structure in the left mediastinum (**e**) originates from the left subclavian artery and ends in the left pulmonary artery (**f**)

Vascular structures located on the left side of the mediastinum in patients with the history of cardiac surgery performed for complex CA may also be included in the differential list of PLSVC. The differential diagnosis could be made by knowing the performed surgery and demonstrating the drainage site of the vessel. Bicaval Glenn shunt, the left-sided Blalock-Taussig (BT) shunt, and collateral vessels after Fontane surgery are possible differentials (Fig. 23).

Bicaval Glenn shunt is an anastomosis of both SVCs to pulmonary arteries in the presence of PLSVC. The Glenn shunt allows the direct drainage of venous blood into the pulmonary arteries via bypassing the right heart chambers [40, 41].

BT shunt is one of the surgical methods for complex CA. In this procedure, the connection of the subclavian artery and the pulmonary artery is enabled via the graft placement [40, 42].

In Fontan surgery, the SVC and IVC are anastomosed to the pulmonary artery. After surgery, collaterals, which may be seen as large vessels on the left side of the mediastinum, may develop and may be confused with PLSVC [43].

## Conclusion

In conclusion, PLSVC is the most common thoracic venous anomaly known to be mostly asymptomatic. However, contrary to common misconception, it may cause a number of clinically significant symptoms, even in a heart with normal anatomy. Likewise, it may significantly affect the proper approaches to heart transplantations, effective surgical treatments for complex cardiac anomalies, and ablative procedures for cardiac arrhythmias. Thus, it should be recognized correctly and reported explicitly in radiological reports, even when it is an incidental finding. Besides, it is important to be aware of differential diagnoses of PLSVC and their radiological features to correctly interpret the vascular structures on the left side of the mediastinum.

## Data Availability

Data sharing is not applicable to this article as no datasets were generated or analyzed during the current study.

## Abbreviations

3D VRT: Three-dimensional volume rendering technique
AF: Atrial fibrillation
ALBV: Aberrant left brachiocephalic vein
ALSA: Aberrant left subclavian artery
APVD: Anomalous pulmonary venous drainage
ARSA: Aberrant right subclavian artery
ASD: Atrial septal defect
AVSD: Atrioventricular septal defect
AzV: Azygos vein
BT shunt: Blalock-Taussig shunt
BV: Bridging vein
CA: Cardiac anomalies
CCV: Common cardinal vein
CHD: Congenital heart disease
CoA: Coarctation of the aorta
CS: Coronary sinus
CTMs: Conotruncal malformations
CV: Cardinal vein
CVC: Central venous catheter
DORV: Double outlet right ventricle
DSVC: Double SVC
IPLSVC: Isolated PLSVC
ITVP: Inferior transverse venous plexus
IVC: Inferior vena cava
JV: Internal jugular vein
L/D-TGA: Levo/dextro-transposition of the great arteries
LA: Left atrium
LACV: Levoatriocardinal vein
LBCV: Left brachiocephalic vein
LICV: Left inferior cardinal vein
LOLs: Left-sided obstructive lesions
LSCV: Left superior cardinal vein
LSICV: Left superior intercostal vein
MDCT: Multidetector computed tomography
MRA: Magnetic resonance angiography
MRI: Magnetic resonance imaging
OV: Oblique vein of the left atrium
PA: Pulmonary atresia
PAPVD: Partial APVD
PCPV: Pericardiophrenic vein
PDA: Patent ductus arteriosus
PLSVC: Persistent left superior vena cava
PS: Pulmonary stenosis
RA: Right atrial structure
RAA: Right aortic arch
RICV: Right inferior cardinal vein
RSCV: Right superior cardinal vein
RSVC: Right superior vena cava
RVOTO: Right ventricular outflow tract obstruction
SCV: Subclavian vein
STVP: Superior transverse venous plexus
SV: Sinus venosus;
SVC: Superior vena cava
TA: Truncus arteriosus
TAPVD: Total APVD
TOF: Tetralogy of Fallot
VSD: Ventricular septal defect
VV: Vertical vein
