# Correction to: Persistent left superior vena cava: clinical importance and differential diagnoses

**DOI:** 10.1186/s13244-021-00983-x

**Published:** 2021-04-16

**Authors:** Aynur Azizova, Omer Onder, Sevtap Arslan, Selin Ardali, Tuncay Hazirolan

**Affiliations:** Department of Radiology, Hacettepe University School of Medicine, 06100 Ankara, Turkey

## Correction to: Insights Into Imaging 11:110 10.1186/s13244-020-00906-2

Unfortunately, this article published on October 15 2020 contains several errors:

1. The references specified in the paragraph “Moreover, attention has been drawn to the relationship of some specific cardiac anomalies with PLSVC in many publications. In addition to left-sided pathologies such as mitral atresia, cor triatriatum, and hypoplastic left heart, transposition of the great arteries (TGA) and tricuspid atresia are other rarer anomalies that have been reported to be closely related to PLSVC in the literature [6, 16, 20].” should be [8, 16, 20].
2. In Table 4, first column containing references is incorrect for all references except for the first one (Perles et al. [1]).  Correct numbering should be as follows:
  Nagasawa et al. [8]
  Lendzian et al. [17]
  Ari et al. [14]
  Berg et al. [15]
  Oztunc et al. [21]
3. In Table 4 and in the second paragraph of the subsection named “PLSVC and accompanying cardiac anomalies,” one reference is incorrectly spelled: Lendzian et al. was mistakenly written as Lendzidan et al.

The original article has been updated.

